# Competition between π‑π and NH···π Interactions in Pyrrole^+^‑Benzene and Pyrrole^+^‑Toluene Radical Cations Revealed by IR Spectroscopy

**DOI:** 10.1021/acs.jpca.5c05818

**Published:** 2025-10-29

**Authors:** Dashjargal Arildii, Otto Dopfer

**Affiliations:** Institut für Physik und Astronomie, 566188 Technische Universität Berlin, Hardenbergstrasse 36, Berlin 10623, Germany

## Abstract

The charge resonance (CR) interaction is among the strongest intermolecular forces in aromatic dimer cations (∼100 kJ mol^–1^). Its strength strongly depends on the ionization energy differences of the two interacting aromatic units (ΔIE). Therefore, it is the strongest in homodimers (A_2_^+^) and forms π-stacked sandwich structures, like in the pyrrole dimer cation (Py_2_^+^). In heterodimers (ΔIE ≠ 0), the CR is weakened, allowing other noncovalent forces, such as cation-π interactions, to compete in strength. Herein, we investigate the binding motifs of the pyrrole^+^-benzene (Py^+^Bz) and pyrrole^+^-toluene (Py^+^Tol) heterodimers, with ΔIE = 1.04 and 0.62 eV, respectively. The NH stretch vibrations (ν_NH_) of mass-selected bare and colder Ar-tagged clusters of Py^+^Bz and Py^+^Tol, recorded by infrared photodissociation spectroscopy and analyzed using dispersion-corrected density functional theory calculations, provide detailed insight into the preferred binding motifs and their relative strengths. For both dimers, NH···π hydrogen bonding (H-bonding) dominates over the CR interaction, favoring T-shaped geometries over π-stacked structures because their large ΔIE values prevent the formation of a strong CR between the two π-systems. The systematic redshifts of ν_NH_ are correlated with the NH···π H-bond strength in Py^+^Bz and Py^+^Tol and thus enable quantitative evaluation of such cation-π interaction energies. A minor population of less stable π-stacked isomers is observed for both Py^+^Bz and Py^+^Tol. Local energy decomposition analysis reveals that the π-stacked structures are not stabilized by a strong CR but rather by weaker π-π stacking interactions governed by electrostatic, induction, and dispersion forces.

## Graphical abstract

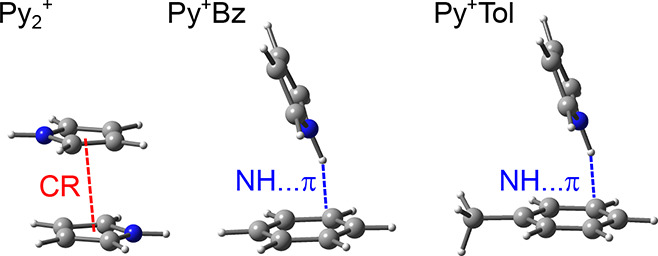

## 1 Introduction

Noncovalent interactions involving aromatic π-electrons, such as π-π stacking and π-hydrogen bonds (π H-bonds) are fundamental for many chemical and biological processes, including protein folding, molecular recognition, molecular assembly, and crystal growth in both liquid and solid phases.^1–17^ In positively charged aromatic aggregates, the cation-π interaction is one of the strongest among the noncovalent interactions due to electrostatic and induction forces between the cation and the electron-rich aromatic π-electron system. Binding energies between metal cations and aromatic molecules, such as benzene, range from 80 to 160 kJ mol^–1^ for alkali metals, depending on their ionic radius.^6,18–20^ These interactions are even stronger for transition metals due to partial charge transfer.^21–24^ The strength of the cation-π interaction also increases with the size of the π-system because of enhanced induction effects.^6,18,25^ In addition to metal cations, the ammonium cation (NH_4_^+^) forms a strong cation-π bond to aromatic molecules comparable to the potassium cation (K^+^), with binding energies of around ∼80 kJ mol^–1^.^26^ Consequently, NH···π H-bonds between protonated amino groups (ammonium) and aromatic residues of amino acids are important cation-π interactions and play a vital role in structural stabilization and folding of proteins.^6,27–30^ Further cation-π interactions between protonated water or methanol clusters and benzene (OH···π H-bonds) have been characterized as model system for cations interacting with a hydrophobic surface.^31,32^

In aromatic dimer cations, charge resonance (CR) interactions are even stronger (∼100 kJ mol^–1^) than their cation-π interactions (∼50–80 kJ mol^–1^)^18,26^ and contribute significantly to structure, energetics, and dynamics of intermolecular and intramolecular stacking interactions, as well as charge transport in conjugated biological and organic semiconductor materials.^33–44^ The CR in aromatic heterodimer cations (AB^+^) is formed by sharing the positive charge, generated by removing a single π electron, between A and B. As a result, the CR leads to a splitting between two electronic states into a stabilized ground electronic state (ψ_+_) and a repulsive excited electronic state (ψ_–_),^33,45–47^ which strongly depends on the difference in the ionization energies (ΔIE) of A and B. The CR becomes stronger with decreasing ΔIE, and thus is strongest for homodimer cations, A_2_^+^ (ΔIE = 0).^35,48^ The rather intense electronic CR transition (ψ_–_ ← ψ_+_), measured for various aromatic homo- and heterodimer cations using optical spectroscopy, occurs typically around 1 μm. It is found to be broad even in the gas phase because it involves excitation into the repulsive ψ_–_ state.^46–52^ Consequently, optical spectra of heterodimer cations provide only a rough estimation of the asymmetric charge distribution present in AB^+^ heterodimers.^45,50,52^ Mass spectrometric studies of heterodimers, such as (benzene-toluene)^+^, (benzene-naphthalene)^+^, and (naphthalene-methylnaphthalene)^+^, demonstrate an inverse correlation between their binding energies and ΔIEs.^53–56^ However, the impact of ΔIE on the dimer structures, along with its correlation to the CR properties, is still not well understood.^35^

Recently, we introduced high-resolution infrared photodissociation (IRPD) spectroscopy as a versatile tool to analyze the charge distribution and geometry changes in isolated aromatic dimers for the prototypical CR-stabilized pyrrole dimer cation (Py_2_^+^) by monitoring the vibrational transitions in the stable ground state (ψ_+_).^35^ Unlike benzene (Bz) and related polycyclic aromatic hydrocarbons, whose CH stretch modes (ν_CH_) are less sensitive to the charge with weaker IR oscillator strengths in the cation ground state,^46,50,57–61^ Py has a single isolated, uncoupled, and strongly IR-active NH stretch oscillator, whose frequency depends rather strongly on its charge state (e.g., ν_NH_ = 3531, 3479, 3447 cm^–1^ for *q*_Py_ = 0, +0.5, +1*e*), resulting in a rather linear correlation between ν_NH_ and *q*_Py_.^35,62,63^ Importantly, Py_2_^+^ exclusively forms the CR-stabilized π-π sandwich structure rather than a T-shaped NH···π H-bonded structure observed for its Py_2_ neutral counterpart,^64,65^ illustrating the large impact of ionization on structure and bonding of aromatic clusters.^7–9^ We have also investigated the influence of symmetry reduction on the CR in Py_2_^+^ by either solvation (e.g., Py_2_^+^L with L = N_2_ or (H_2_O)_1–3_) or by substitution of functional groups (e.g., Py^+^X with X = *N*-methyl-Py).^35,66,67^ In both cases, the perturbation of the symmetry of the CR in Py_2_^+^ remains rather modest, resulting in only small changes in the charge distribution of the free Py unit (Δ*q*_Py_ ≤ 87 m*e*).^35,66,67^ In solvated Py_2_^+^L clusters, the CR appears to be substantially stronger than the perturbations caused by the noncovalent interactions with the ligands. On the other hand, the CR in Py^+^X heterodimers, where one Py unit in Py_2_^+^ is replaced by a different aromatic molecule X with a substantially different IE (i.e., large ΔIE), is expected to be strongly affected (i.e., weakened) due to the pronounced redistribution of the excess charge. As a result, other intermolecular binding motifs, such as π-π stacking or π H-bonding, may become competitive with the reduced CR interaction.^68,69^

To this end, we characterize herein the Py^+^X heterodimers with X = benzene (Bz) and toluene (Tol) using IRPD of mass-selected bare and Ar-tagged clusters in the CH and NH stretch ranges (ν_CH/NH_). Complementary dispersion-corrected density functional theory (DFT) calculations are employed to assign the measured IRPD spectra and to analyze the observed intermolecular interactions and charge distributions. Bz and Tol have significantly higher IEs of 9.244 and 8.828 eV than Py (8.207 eV).^70^ The resulting large ΔIE values of 1.04 and 0.62 eV, respectively, will cause a substantial reduction of the strong CR present in Py_2_^+^.^35^ Hence, π-π stacking and π H-bonds may become competitive with the CR. Thus, we aim to systematically characterize the changes in interaction type within the Py^+^X heterodimers (including geometry and binding motif, binding energy, charge redistribution) as a function of ΔIE. In particular, Py^+^ has a strongly acidic NH H-bond donor group, which enables an NH···π (cation-π) H-bonding motif. A similar NH···π interaction has been observed for the neutral PyBz counterpart.^71^ No experimental data appear to be available for neutral and cationic Py^(+)^Tol dimers.

## 2 Experimental and Computational Details

IRPD spectra of mass-selected Py^+^X and Py^+^XAr (X = Bz and Tol) cluster cations are measured in a tandem quadrupole mass spectrometer coupled to an electron ionization source and an octupole ion guide.^9,72^ The cluster cations are produced in a pulsed supersonic plasma expansion of Py (Sigma-Aldrich, >98%) and Bz (Fluka, ≥99.5%) or Tol (Sigma-Aldrich, ≥99.5%), seeded in Ar carrier gas (7 bar, nozzle temperature 50 °C). Ionic clusters are formed by electron and/or chemical ionization and subsequent three-body aggregation reactions. The cluster cations of interest are selected by the first quadrupole mass filter and irradiated in the adjacent octupole with a tunable IR laser pulse (ν_IR_) emitted from an optical parametric oscillator and amplifier pumped by a Q-switched nanosecond Nd:YAG laser. The IR radiation is characterized by a pulse energy of 2–5 mJ, a bandwidth of <2 cm^–1^, and a repetition rate of 10 Hz. The IR laser frequency is calibrated to better than 1 cm^–1^ using a wavemeter. Resonant vibrational excitation induces the loss of the most weakly bonded ligand (i.e., Ar, Bz, or Tol). The generated fragment ions are selected by the second quadrupole mass filter and monitored by a Daly detector as a function of ν_IR_ to obtain the IRPD action spectrum of the parent cluster. The ion source is triggered at twice the laser frequency to subtract the metastable decay background from the total fragment ion signal for extracting the laser-induced dissociation signal. All IRPD spectra are linearly normalized for frequency-dependent variations in the IR photon flux measured by a pyroelectric detector. The peak widths of the vibrational transitions observed in the IRPD spectra are mainly caused by unresolved rotational structure, sequence hot bands involving inter- and intramolecular modes, and possible contributions of several isomers. In addition to laser-induced dissociation (LID and IRPD), low-energy collision-induced dissociation (CID) experiments are performed in the octupole ion guide to confirm the composition of the mass-selected parent clusters. For recording CID mass spectra, the octupole is filled with air (10^–5^ mbar), resulting in ion-neutral collisions with 10 eV energy in the laboratory frame.

Quantum chemical calculations are carried out at the dispersion-corrected CAM-B3LYP-D3/aug-cc-pVTZ level to determine the structural, energetic, vibrational, and electronic properties of the investigated cluster cations.^73–77^ Energies of the optimized structures obtained using the PBE0-D3 ^78^ and M06–2X-D3 ^79^ and CBS-QB3 ^80,81^ approaches are employed to evaluate the reliability of the computational results at the CAM-B3LYP-D3 level. Harmonic vibrational frequencies are scaled by a factor of 0.9530 for NH and CH stretch vibrations to optimize the agreement with the measured frequency of Py^+^ (ν_NH_ = 3447 cm^–1^).^62^ Intermolecular interaction energies (*D*_0_) and relative energies (*E*_0_) are corrected for harmonic zero-point vibrational energy. Relative free energies (*G*_0_) are calculated at 298 K. The charge distributions on each molecular component are obtained employing the natural bond orbital (NBO) analysis.^82^ Potential energy surfaces for isomerization are computed at the CAM-B3LYP-D3/aug-cc-pVTZ level using the nudged elastic band method implemented in the ORCA software package.^83,84^ The intermolecular interactions are analyzed using the noncovalent interaction (NCI) approach, and details are given in the Supporting Information. Individual contributions to the intermolecular interactions are evaluated using the local energy decomposition (LED) approach at the DLPNO-CCSD­(T)/def2-SVP level.^83–85^ Cartesian coordinates of all considered isomers and their corresponding energies are provided in the Supporting Information.

## 3 Results and Discussion

The IRPD spectra of Py^+^Bz­(Ar) and Py^+^Tol­(Ar) shown in Figure 1 are recorded in the range of 2700–3600 cm^–1^ and cover the free and bound NH stretch modes, ν_NH_^f^ (E) and ν_NH_^b^ (F), the aromatic CH stretch modes, ν_CH_ (G), and the overtone of the NH bending mode, 2β_NH_ (Y).^86^ For comparison, the position of ν_NH_^f^ of bare Py^+^ (3447 cm^–1^)^62^ is indicated by a gray dotted line. The positions and widths of the transitions observed are compiled in Table 1 and Table S1, along with the suggested vibrational and isomer assignments.

**Table 1:** Positions (in cm^–1^) and Suggested Vibrational Assignments of the NH Stretch Transitions Observed in the IRPD Spectra of Py^+^Bz­(Ar) and Py^+^Tol­(Ar) Compared to Frequencies of the Most Stable Isomers Calculated at the CAM-B3LYP-D3/aug-cc-pVTZ Level

	exp^a^	isomer	calc^b^	mode
Py^+^Bz	F 3183 (73)	Py^+^Bz(I)	3239 (945)	ν_NH_^b^
	E 3470 (nr)	Py^+^Bz(II)	3474 (202)	ν_NH_^f^
Py^+^BzAr	F 3188 (24)	Py^+^Bz(I)Ar(I)	3238 (983)	ν_NH_^b^
Py^+^Tol	F 3150 (63)	Py^+^Tol(I)	3215 (982)	ν_NH_^b^
	E 3450 (nr)	Py^+^Tol(II)	3449 (89)	ν_NH_^f^
Py^+^TolAr	F 3159 (38)	Py^+^Tol(I)Ar(I)	3231 (948)	ν_NH_^b^

a Peak positions and widths (in parentheses) are obtained from deconvolution. nr = not resolved, broad.

b IR intensities in parentheses are given in km mol^–1^.

**Figure 1:**
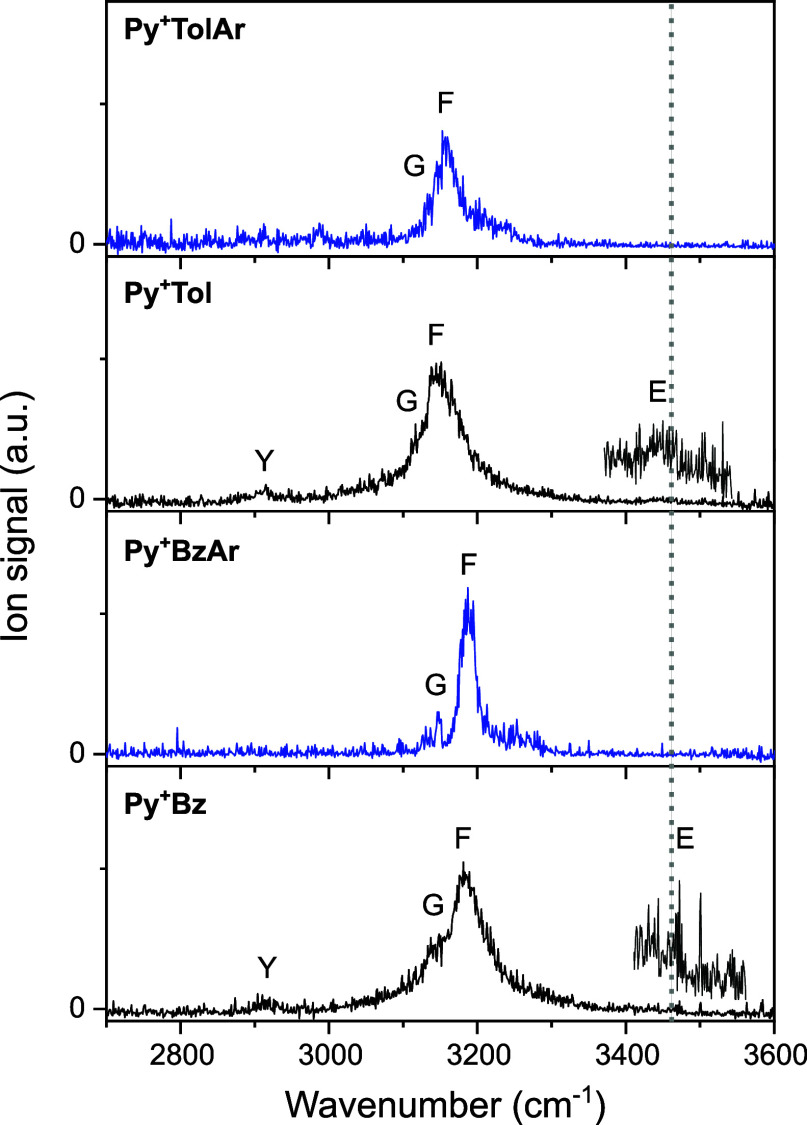
IRPD spectra of Py^+^Bz­(Ar) and Py^+^Tol­(Ar) recorded in the CH and NH stretch range. The spectra of Py^+^Bz and Py^+^Tol are magnified by a factor of 10 in the 3400–3550 cm^–1^ range. The positions, widths, and assignments of the transitions observed are compiled in Table 1 and Table S1. For comparison, the ν_NH_^f^ frequency of bare Py^+^ (3447 cm^–1^) is indicated by a gray dotted line.

### 3.1 Py^+^Bz

The IRPD spectrum of Py^+^Bz exhibits three major peaks F, G, and Y at 3183, 3148, and 2915 cm^–1^, respectively, with widths of 25–73 cm^–1^, and one minor peak E observed at 3470 cm^–1^. The largely red-shifted (−264 cm^–1^) and rather intense ν_NH_^b^ band (F) with its blueshaded contour suggests the presence of an H-bonded isomer with a strong NH···π H-bond, whereas the slightly blueshifted (+23 cm^–1^) and very weak ν_NH_^f^ band (E) indicates the scarce contribution of a dimer with a free NH group. To confirm the structural and vibrational assignments, the IR spectra computed for the low-energy structures of Py^+^Bz (Figure 2) are compared in Figure 3a to the measured IRPD spectrum (Table 1 and Tables S1 and S2).

**Figure 2:**
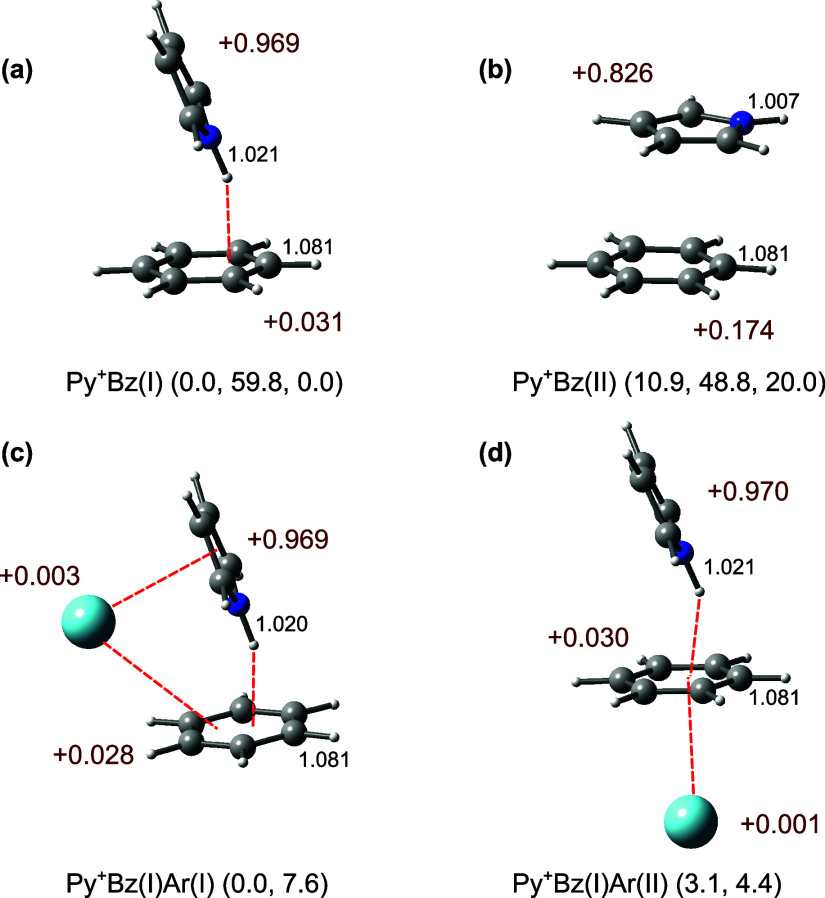
Structures of (a, b) Py^+^Bz­(I–II) and (c, d) Py^+^Bz­(I)­Ar­(I–II) obtained at the CAM-B3LYP-D3/aug-cc-pVTZ level. Selected intramolecular bond lengths (in Å) are given in black. Values in dark red indicate NBO charges (in units of *e*). Energies in parentheses are relative energy, dissociation energy of the most weakly bonded ligand, and relative Gibbs free energy at 298 K (*E*_0_, *D*_0_, and *G*_0_ in kJ mol^–1^). *D*_0_ accounts for the loss of Bz for panels (a, b) and Ar for panels (c, d), respectively.

**Figure 3:**
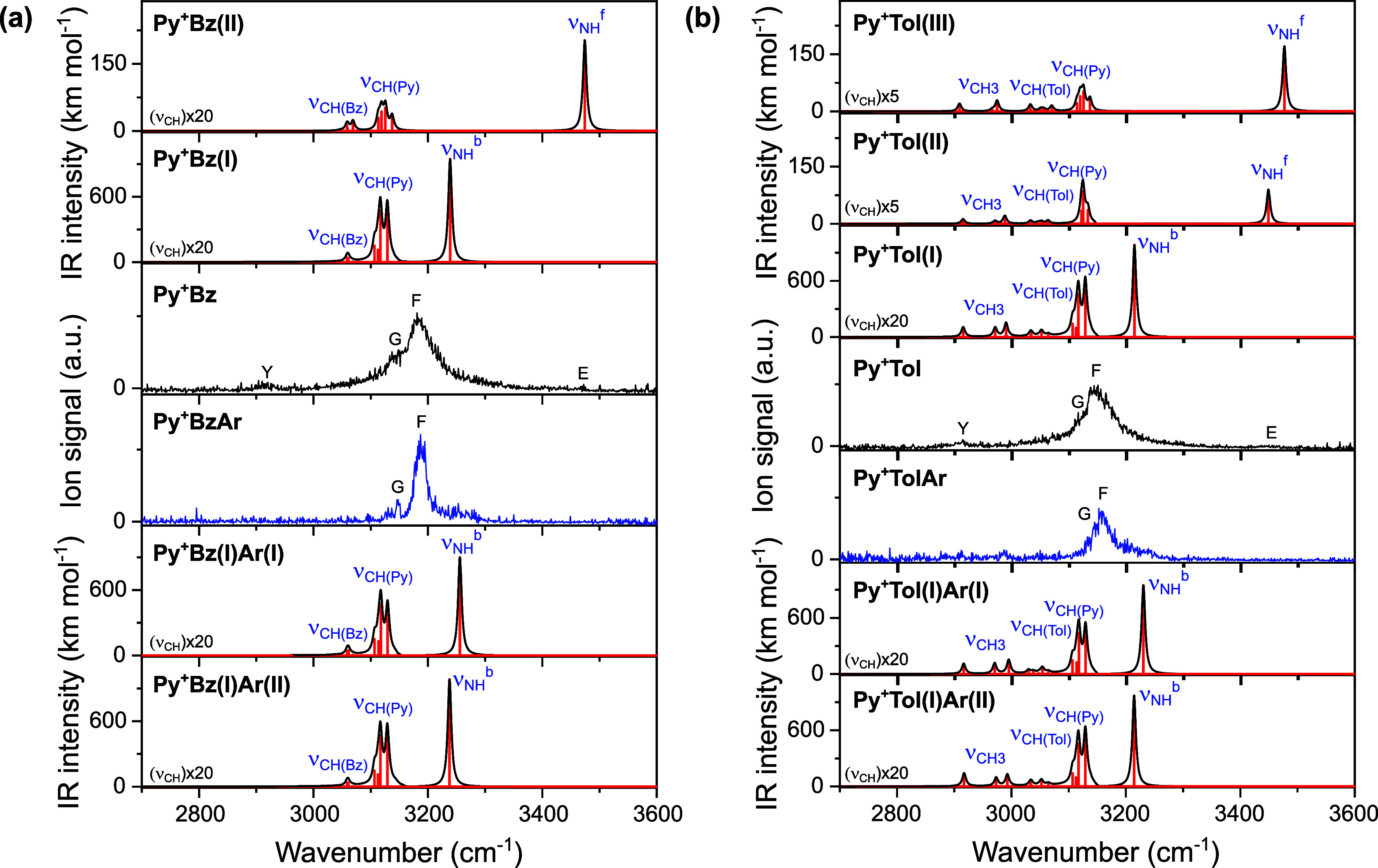
IRPD spectra of (a) Py^+^Bz­(Ar) compared to linear IR absorption spectra calculated for Py^+^Bz­(I–II) and Py^+^Bz­(I)­Ar­(I–II) and (b) Py^+^Tol­(Ar) compared to linear IR absorption spectra calculated for Py^+^Tol­(I–III) and Py^+^Tol­(I)­Ar­(I–II) in the CH and NH stretch range at the CAM-B3LYP-D3/aug-cc-pVTZ level (Table 1 and Table S1). For better visibility, the intensities of the CH stretch bands are multiplied by factors of 20 or 5, respectively.

In the H-bonded Py^+^Bz­(I) global minimum with *C*_s_ symmetry, Bz binds to the NH group of Py^+^ via a strong NH···π H-bond with *D*_0_ = 59.8 kJ mol^–1^ (Figure 2a). The NH group of Py^+^ points toward one of the C atoms of Bz, resulting in six equivalent minima on the potential arising from hindered rotation of Bz around its *C*_6_ axis. As a result, the N–H bond elongates by 11 mÅ to 1.021 Å (see Figure S1 for the structures of bare Py^(+)^), which leads to a computed redshift of −208 cm^–1^ for ν_NH_ consistent with the experimental shift of −264 cm^–1^. As expected, the ν_CH_ modes of both Py and Bz are barely affected. The NBO charges reveal that the positive charge is mainly localized on Py (+0.969*e* vs +0.031*e*) because of its lower IE compared to Bz (8.207 vs 9.244 eV),^70^ justifying the notation of Py^+^Bz. The π-stacked Py^+^Bz­(II) isomer (Figure 2b) is predicted to be significantly less stable than Py^+^Bz­(I) by Δ*E*_0_ = 10.9 kJ mol^–1^ (*D*_0_ = 48.8 kJ mol^–1^). The π-π interaction present in Py^+^Bz­(II) is substantially weaker than a typical CR interaction (∼100 kJ mol^–1^)^33–35^ because of the large ΔIE of 1.04 eV. Instead, it is mostly based on long-range electrostatic, induction, and dispersion forces. The N–H bond of Py^+^ in Py^+^Bz­(II) contracts slightly (by 3 mÅ) because of the partial charge transfer to Bz (+0.174*e*), leading to a small but noticeable blueshift of +27 cm^–1^ for ν_NH_^f^ (3474 vs 3447 cm^–1^), in close agreement with the experimental observation (+23 cm^–1^). Interestingly, the *E*_0_ values of Py^+^Bz­(II) predicted at the PBE0-D3 (3.3 kJ mol^–1^), M06–2X-D3 (6.6 kJ mol^–1^), and CBS-QB3 (10.8 kJ mol^–1^) levels are even lower than for CAM-B3LYP-D3 (10.9 kJ mol^–1^, Table S3), which suggests a tighter competition between the π-π and NH···π binding motifs. However, at higher temperatures the π-stacked structure becomes far less stable than the H-bonded one (Δ*G*_0_ = 20.0 kJ mol^–1^), confirming that the π-π interaction in Py^+^Bz­(II) is indeed weaker than the NH···π H-bond of Py^+^Bz­(I) also at elevated temperatures.

The IRPD spectrum of Py^+^Bz is compared in Figure 3a to the IR spectra computed for the two most stable isomers. Peaks F and G at 3183 and 3148 cm^–1^ are readily assigned to ν_NH_^b^ and ν_CH_ of Py^+^Bz­(I), considering the largely red-shifted ν_NH_^b^ resulting from the formation of the NH···π H-bond. Peak Y is attributed to the overtone of the NH bending mode of isomer I (2β_NH_ at 2934 cm^–1^, scaled by a factor of 0.98), which gains its intensity from a Fermi resonance (FR) with the nearby intense ν_NH_^b^ fundamental. The barely discernible peak E suggests a rather minor contribution of π-stacked Py^+^Bz­(II), indicating that the observed population is clearly dominated by the H-bonded Py^+^Bz­(I) global minimum under our experimental conditions. The abundance of isomer II estimated from the relative intensity of peak E with respect to F (1:100) and the corresponding computed IR oscillator strengths (1:5) is of order ∼5%. This value is consistent with the prediction of the thermodynamic equilibrium population ratio (∼7%) derived from a Boltzmann distribution at 298 K and the computed *E*_0_ values.

The IRPD spectrum of Ar-tagged Py^+^Bz displays two major peaks G and F at 3147 and 3188 cm^–1^ with narrower widths of 12 and 24 cm^–1^ and almost negligible Ar shifts of −1 and +5 cm^–1^, respectively. Thus, Ar-tagging has only a minor effect on the most abundant cluster, while providing a colder and thus better resolved IRPD spectrum. Significantly, the absence of peak E in the IRPD spectrum of Py^+^BzAr indicates that the population of the Ar-tagged π-stacked Py^+^Bz­(II) isomer, estimated from the achieved signal-to-noise ratio (35:1), is below the detection limit (<15%). Ar has only a small effect on structures, relative energies, and IR intensities. Thus, the absence of the Py^+^Bz­(II) isomer in the IRPD spectra of the tagged species is mainly attributed to more efficient cooling. Therefore, only the Ar-tagged isomers of Py^+^Bz­(I) are experimentally observed and considered further (Figure 2). In the computed global minimum, Py^+^Bz­(I)­Ar­(I), Ar binds to the π-ring of Py^+^ with *D*_0_ = 7.6 kJ mol^–1^. Due to steric effects arising from the Ar···Bz contact, Ar-tagging weakens the NH···π H-bond between Py^+^ and Bz. Therefore, the N–H bond of Py^+^ becomes slightly shorter (less elongated by 1 mÅ) compared to that of bare Py^+^Bz­(I), causing a small blueshift of +17 cm^–1^ for ν_NH_^b^, consistent with the experimental observation. In Py^+^Bz­(I)­Ar­(II), which is less stable than Py^+^Bz­(I)­Ar­(I) by 3.1 kJ mol^–1^ at 0 K, Ar binds only to the π-ring of Bz with *D*_0_ = 4.4 kJ mol^–1^. Because Ar-tagging in Py^+^Bz­(I)­Ar­(II) has a negligible effect on the N–H bond of Py^+^, ν_NH_^b^ is nearly unaffected (3238 cm^–1^), in disagreement with experiment. For completeness, further higher energy isomers of π-stacked Py^+^Bz­(II)Ar are shown in Figure S2. Because they are not detected in the IRPD spectrum (Figure S3), they are not discussed further here. In conclusion, the comparison between the IRPD spectrum measured for Py^+^BzAr and the IR spectra computed for the most stable isomers suggests an assignment to the Py^+^Bz­(I)­Ar­(I) global minimum. While the Ar binding site is less certain, the IRPD spectrum of the Ar-tagged Py^+^Bz ions clearly confirms the predominant presence of the most stable H-bonded Py^+^Bz­(I) isomer.

The minimum energy path on the potential energy surface for converting the T-shaped Py^+^Bz­(I) global minimum into the π-stacked Py^+^Bz­(II) local minimum by hindered internal rotation is determined using the nudged elastic band method implemented in the ORCA software package (Figure 4a).^83,84^ The relative energies of Py^+^Bz­(I) and Py^+^Bz­(II) of *E*_e_ = 0 and 9.7 kJ mol^–1^ are comparable to *E*_0_ = 0 and 10.9 kJ mol^–1^, which are corrected for zero-point vibrational energy. The internal rotation barriers are *V*_b_(I→II) = 13.1 and *V*_b_(II→I) = 3.4 kJ mol^–1^ for the forward and backward reaction, respectively.

**Figure 4:**
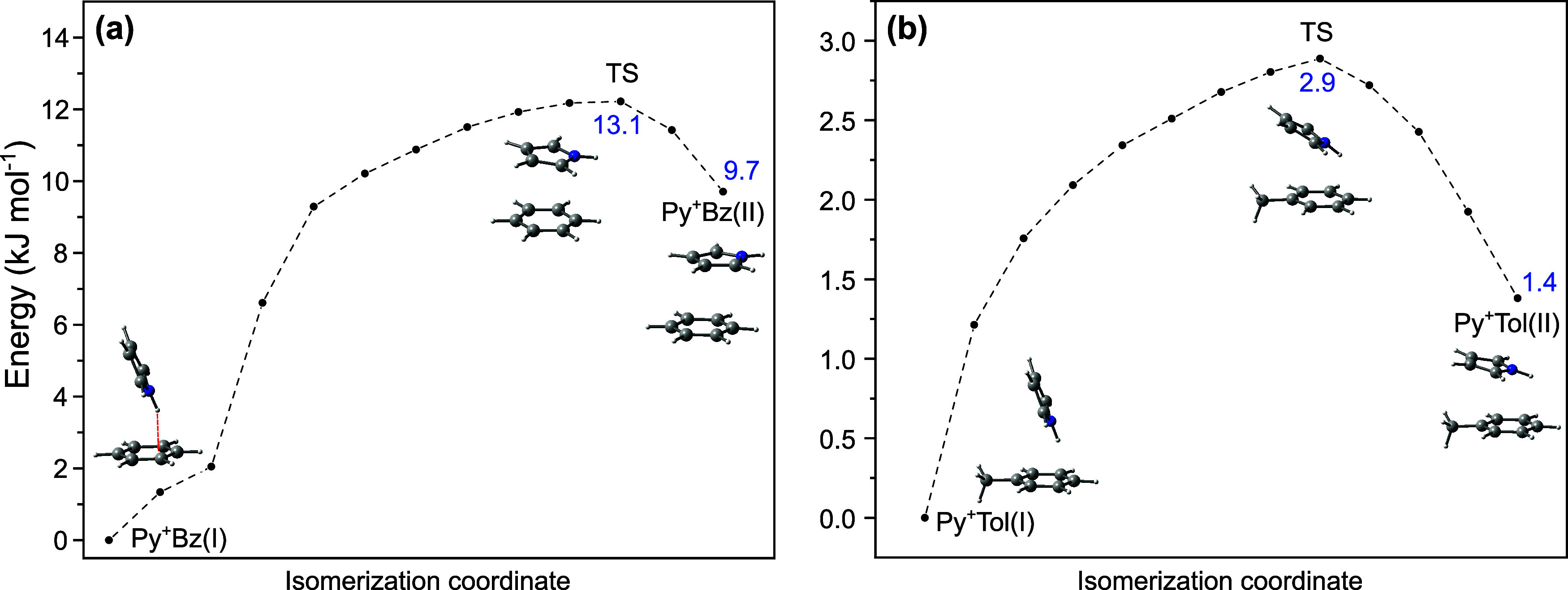
Potential energy surface (uncorrected for zero-point vibrational energy) for the isomerization (a) Py^+^Bz­(I) ↔ Py^+^Bz­(II) and (b) Py^+^Tol­(I) ↔ Py^+^Tol­(II) using the nudged elastic band method (CAM-B3LYP-D3/aug-cc-pVTZ).

### 3.2 Py^+^Tol

The IRPD spectrum of Py^+^Tol exhibits peaks E, F, G, and Y at 3450, 3150, 3128, and 2914 cm^–1^, respectively. The Py^+^Tol spectrum is rather similar in appearance to the Py^+^Bz spectrum, suggesting an analogous vibrational and isomer assignment. The ν_NH_^b^ band (F) of the H-bonded isomer of Py^+^Tol exhibits a slightly larger redshift compared to Py^+^Bz (−297 vs −264 cm^–1^), indicative of a somewhat stronger and shorter NH···π H-bond. The weak ν_NH_^f^ band (E) suggests again a minor contribution of a π-stacked isomer with a free NH group. The IR spectra computed for the optimized structures of Py^+^Tol (Figure 5) are compared in Figure 3b to the measured IRPD spectrum (Table 1 and Table S1).

**Figure 5:**
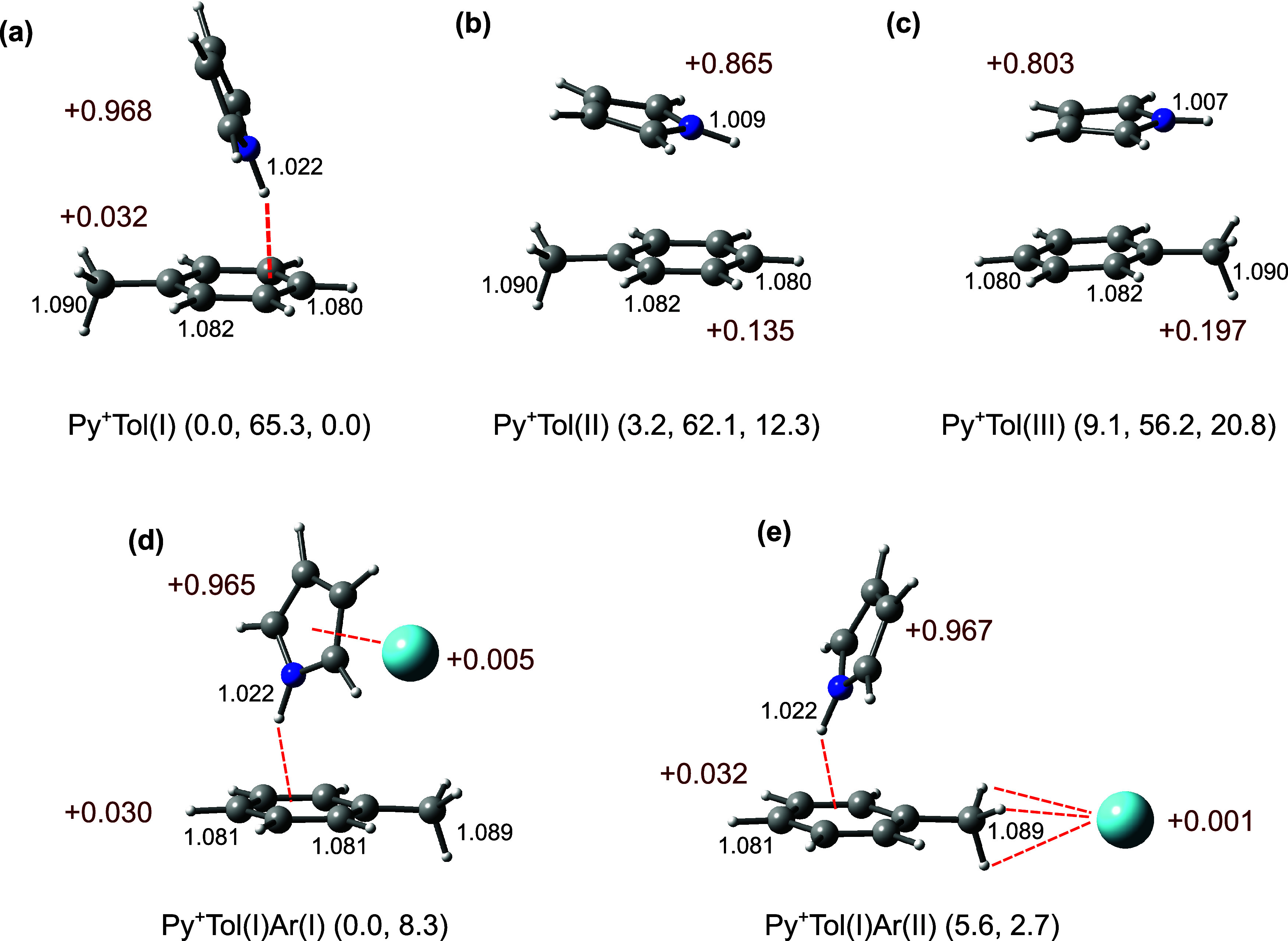
Structures of (a–c) Py^+^Tol­(I–III) and (d, e) Py^+^Tol­(I)­Ar­(I–II) obtained at the CAM-B3LYP-D3/aug-cc-pVTZ level. Selected intramolecular bond lengths (in Å) are given in black. Values in dark red indicate NBO charges (in units of *e*). Energies in parentheses are relative energy, dissociation energy of the most weakly bonded ligand, and relative Gibbs free energy at 298 K (*E*_0_, *D*_0_, and *G*_0_ in kJ mol^–1^). *D*_0_ accounts for the loss of Tol for panels (a–c) and Ar for panels (d, e), respectively.

In the T-shaped global minimum with *C*_s_ symmetry, Py^+^Tol­(I), Tol binds to the NH group of Py^+^ via an NH···π H-bond with *D*_0_ = 65.3 kJ mol^–1^ (Figure 5a). Indeed, this NH···π H-bond is calculated to be slightly stronger than that of Py^+^Bz­(I) by 5.5 kJ mol^–1^. This increase in binding energy arises from the additional permanent electric dipole moment of Tol (0.375 D)^70^ and its larger polarizability compared to Bz (11.861 vs 9.959 Å^3^),^70^ which enhance electrostatic, induction, and dispersion forces. The NCI analysis further supports the relative H-bond strengths, with ρ* values of −0.020 and −0.019 au for Py^+^Tol and Py^+^Bz, respectively (Figure S4). Therefore, the N–H bond of Py^+^ elongates even more compared to that of Py^+^Bz, resulting in a larger predicted redshift of ν_NH_^b^ (−232 vs −208 cm^–1^), in agreement with the experimental observation. Again, the ν_CH_ modes of Py^+^ and Tol are barely affected by the formation of the NH···π H-bond. Similar to Py^+^Bz­(I), the positive charge in Py^+^Tol­(I) is largely located on Py (+0.968*e* vs +0.032*e*) because the IE of Py is still substantially lower than that of Tol (8.207 vs 8.828 eV). Py^+^Tol­(II) and Py^+^Tol­(III) are π-stacked isomers at only slightly higher energies, *E*_0_ = 3.2 and 9.1 kJ mol^–1^, respectively (Figure 5b,c). In Py^+^Tol­(II) with *C*_s_ symmetry and *D*_0_ = 62.1 kJ mol^–1^, the NH group of Py^+^ is aligned antiparallel to the CH_3_ group of Tol, because electrostatic forces (charge–dipole and dipole–dipole) favor such an orientation. However, although its binding energy is higher than for Py^+^Bz­(II) (62.1 vs 48.8 kJ mol^–1^), it is still significantly lower than a typical CR interaction because ΔIE of this heterodimer is still too large for developing a strong CR. The Py unit in Py^+^Tol­(II) is somewhat tilted toward Tol due to the attraction between the NH group of Py and the π-cloud of Tol. The NBO charge on Py is reduced to +0.865*e* compared to Py^+^Tol­(I) due to the positive charge delocalization into Tol (+0.135*e*) via the π-π interaction. However, the charge delocalization into Tol is lower than into Bz (+0.135*e* vs +0.174*e*) due to the impact of the NH···π interaction on the π-stacked geometry of Py^+^Tol­(II). The NCI analysis reveals an additional weak intermolecular NH···π bond for Py^+^Tol (II) (ρ*=-0.004 au, Figure S5). Therefore, the N–H bond in Py^+^ of Py^+^Tol­(II) contracts only slightly (1 mÅ), leading to a small blueshift of ν_NH_^f^ by +2 cm^–1^ (3449 vs 3447 cm^–1^). In Py^+^Tol­(III), the NH group of Py^+^ is oriented parallel to the CH_3_ group of Tol, which allows better overlap between the π-rings of Py and Tol, resulting in an increased charge delocalization into Tol (+0.197*e*). Therefore, the N–H bond of Py in Py^+^Tol­(III) contracts slightly more than in Py^+^Tol­(II) (3 mÅ), leading to a larger blueshift in ν_NH_^f^ by +30 cm^–1^ (3477 vs 3447 cm^–1^). The competition between the π-stacked and T-shaped Py^+^Tol isomers becomes even tighter compared to Py^+^Bz, because (*i*) the permanent dipole moment of Tol increases the NH···π H-bond strength and (*ii*) the smaller ΔIE of the Tol/Py pair (0.62 eV) also favors the π-orbital overlap, which may indicate the onset of the CR interaction. The total effect leads to a decrease in the energy gap between isomers I and II when replacing Bz by Tol (see Figure S6 for an energy diagram comparing the various isomers of Py^+^Bz and Py^+^Tol).

The IRPD spectrum of Py^+^Tol is compared in Figure 3b to the IR spectra computed for the most stable isomers (I–III). Peak F at 3150 cm^–1^ is readily assigned to ν_NH_^b^ of Py^+^Tol­(I), considering the largely red-shifted ν_NH_^b^ mode of Py^+^Tol­(I) resulting from its strong NH···π H-bond. The observed redshift of −297 cm^–1^ is compatible with the computed value (−232 cm^–1^). The small peak E at 3450 cm^–1^ is attributed to ν_NH_^f^ of a π-stacked isomer, here Py^+^Tol­(II), and the measured blueshift of +3 cm^–1^ agrees well with the computed value of +2 cm^–1^. An assignment of band E to ν_NH_^b^ of Py^+^Tol­(III) may be excluded for its spectral mismatch and its higher energy. Peak G arises from unresolved aromatic ν_CH_ modes of both Py^+^ and Tol of Py^+^Tol­(I). Much weaker bands predicted in the 2900–3000 cm^–1^ range are attributed to the aliphatic CH stretch bands of the CH_3_ group of Tol and may overlap with the 2β_NH_ overtone of isomer I (peak Y), gaining its intensity from a FR with the nearby ν_NH_^b^ fundamental. Similar to Py^+^Bz, the NH···π H-bonded Py^+^Tol­(I) global minimum is predominantly produced in the supersonic plasma expansion under the employed experimental conditions. From the relative integrated intensities of peaks E and F (1:17) and the corresponding computed ν_NH_ oscillator strength ratio (1:11), the abundance of Py^+^Tol­(II) is roughly estimated as ∼40%, which is consistent with the Boltzmann estimate at 298 K derived from the *E*_0_ values (∼28%).

The IRPD spectrum of Ar-tagged Py^+^Tol exhibits two major transitions F and G at 3159 and 3132 cm^–1^, respectively. Ar-tagging has only a small effect on the geometries of the most abundant structures and yields a colder and thus better resolved IRPD spectrum. Similar to Py^+^BzAr, the absence of peak E for Py^+^TolAr indicates that the population of π-stacked Ar-tagged Py^+^Tol isomers (II or III) are below the detection limit (<30%) at the achieved signal-to-noise ratio. Therefore, only Ar-tagged isomers of the observed T-shaped Py^+^Tol­(I) global minimum are discussed in more detail further (Figure 5d,e). For completeness, the Ar-tagged isomers of the Py^+^Tol­(II–III) and their IR spectra are presented in Figures S7 and S8, respectively.

Similar to Py^+^Bz­(I)­Ar­(I), Ar binds in the Py^+^Tol­(I)­Ar­(I) global minimum to the π-ring of Py^+^, only with a slightly stronger bond, *D*_0_ = 8.3 kJ mol^–1^, which weakens the NH···π H-bond between Py^+^ and Tol. This effect increases ν_NH_^b^ by 16 cm^–1^. Py^+^Tol­(I)­Ar­(II), in which Ar binds to the methyl group of Tol with *D*_0_ = 2.7 kJ mol^–1^, is far less stable than Py^+^Tol­(I)­Ar­(I) by 5.6 kJ mol^–1^ at 0 K. Because Ar-tagging has a negligible effect on the N–H bond of Py^+^, ν_NH_^b^ remains nearly the same (3214 cm^–1^). For completeness, structures, IR spectra, and energies of the higher energy Py^+^Tol­(I)­Ar­(III–V) isomers are provided in the Supporting Information (Figures S7 and S8 and Table S4).

The IRPD spectrum of Py^+^TolAr is compared in Figure 3b to the IR spectra computed for Py^+^Tol­(I)­Ar­(I–II). Similar to the IRPD spectrum of Py^+^BzAr, peak F is narrower and slightly blueshifted, indicating that Ar reduces the NH···π H-bond strength by providing additional steric strain in Py^+^Tol. Peak F is attributed to ν_NH_^b^ of Py^+^Tol­(I)­Ar­(I–II), whereby contributions of isomer I will dominate because of its significantly larger stabilization energy. Peak G is assigned to aromatic ν_CH_ modes of Py^+^ and Tol as discussed for bare Py^+^Tol. Much weaker bands visible in the 2900–3000 cm^–1^ range are attributed to the aliphatic CH stretch bands of the CH_3_ group of Tol.

The minimum energy path on the potential energy surface for converting the T-shaped Py^+^Tol­(I) global minimum into the stacked Py^+^Tol­(II) local minimum is shown in Figure 4b. The relative energies of Py^+^Tol­(I) and Py^+^Tol­(II) are *E*_e_ = 0 and 1.4 kJ mol^–1^ (*E*_0_ = 0 and 3.2 kJ mol^–1^), and the internal rotation barriers for the forward and backward reactions are *V*_b_(I→II) = 2.9 and *V*_b_(II→I) = 1.5 kJ mol^–1^, respectively. Overall, this isomerization potential is much flatter than for Py^+^Bz and may explain the high population of the Py^+^Tol­(II) local minimum observed experimentally, as the untagged clusters are not cooled to very low temperatures in the supersonic plasma expansion, and the energy difference between isomers I and II is rather small.

### 3.3 Competition between π-π and NH···π Interactions

Similar to neutral PyBz,^71,87^ the cationic Py^+^Bz­(I) global minimum features a NH···π H-bond, i.e., ionization retains the binding motif of the most stable isomer. However, due to the excess positive charge on Py^+^, the NH···π H-bond becomes about three times stronger in the cation (*D*_0_ = 59.8 vs 18.8 kJ mol^–1^, CAM-B3LYP-D3) because the predominant attraction changes from dipole–quadrupole to charge–quadrupole interaction. As a result, the N–H bond of Py^+^ in Py^+^Bz­(I) elongates by 11 mÅ in Py^+^Bz­(I), while the N–H bond of Py in PyBz stretches only by 2 mÅ (Figure S1), resulting in a significantly larger measured redshift of ν_NH_^b^ in the cation dimer (Δν_NH_^b^ = −264 vs −59 cm^–1^).^71^ On the other hand, for homodimers of aromatic molecules with acidic proton donor groups (e.g., NH), such as Py_2_ and Py_2_^+^, the binding motifs drastically change by ionization from NH···π H-bonding to the CR-stabilized π-stacked structure.^35,64–66,88^ For the Py^+^Bz heterodimer, however, the large ΔIE of Py and Bz (1.04 eV) strongly limits the efficient frontier MO overlap and thus the CR interaction. As a result, only much weaker electrostatic, induction, and dispersion-driven forces can contribute to the π-π stacking interaction between Py^+^ and Bz in Py^+^Bz­(II), leading to much a lower binding energy compared to the strong CR in Py_2_^+^ (*D*_0_ = 48.8 vs 107.4 kJ mol^–1^).^35,66^ The NH···π H-bond in Py^+^Bz­(I) is stronger and becomes favored over the π-π interaction in Py^+^Bz­(II) by Δ*E*_0_ = 10.9 kJ mol^–1^. Although ΔIE is reduced for Py^+^Tol to 0.62 eV, the CR still does not form efficiently, and the NH···π H-bonded T-shaped structure remains more stable and dominant in the experiment. Moreover, the NH···π H-bond becomes stronger (*D*_0_ = 65.3 kJ mol^–1^) due to the enhanced electrostatic and induction forces arising from the additional dipole moment of Tol and its larger polarizability compared to Bz.

In an effort to quantify the individual contributions to the intermolecular interactions, we consider the LED approach,^85,89^ and the results are compiled in Table 2 for Py_2_^+^ and the two isomers of Py^+^Bz and Py^+^Tol. Significantly, the *D*_e_ values obtained at the CCSD­(T) level are in close agreement with those calculated using the CAM-B3LYP-D3 level, confirming the reliability of the chosen DFT level. In general, both the dominant electrostatic and induction contributions (*E*_es/ind_) and the much weaker dispersion energies (*E*_disp_) are larger for the isomers of Py^+^Tol than for those of Py^+^Bz, due to the additional dipole moment of Tol and its larger polarizability. For the NH···π H-bonded Py^+^Bz­(I) and Py^+^Tol­(I) isomers, *E*_es/ind_ and repulsion (*E*_rep_) contribute ∼65% to the total interaction energy (*E*_int_), while dispersion (*E*_disp_) and the triples correction (*E*_T_) are weaker but still significant (∼35%). *E*_int_ of Py^+^Tol­(I) is larger than that of Py^+^Bz­(I) by 5 kJ mol^–1^, reflecting the enhanced *E*_es/ind_ and *E*_disp_ contributions. For the π-stacked Py^+^Bz­(II) and Py^+^Tol­(II) isomers, *E*_disp_ rises to around 50% of *E*_int_, suggesting that dispersion is more important for π-π stacking than for H-bonding due to the closer proximity of the two interacting π-electron clouds. Finally, the Py_2_^+^ homodimer is stabilized by a strong CR and features much larger *E*_rep_ and *E*_es/ind_ contributions (roughly by 1 order of magnitude) than both the H-bonded and π-stacked isomers of the Py^+^Tol and Py^+^Bz heterodimers, due to the shorter distance between the two monomers and the resulting increased orbital overlap. As a consequence of its stronger total interaction, the relative contribution of dispersion is smaller (*E*_disp_/*E*_int_ = 36%). Overall, the LED analysis confirms that the π-π stacking interactions in the heterodimers are predominantly driven by electrostatic, induction, and dispersion forces, rather than by a CR.

**Table 2:** Summary of the Contributions to the Intermolecular Interaction Obtained from the LED Analysis at the DLPNO-CCSD­(T)/def2-SVP Level (in kJ mol^–1^)­^a^

	*E*_rep_	*E*_es/ind_	*E*_disp_	*E*_T_	*E*_int_	*D*_e_ CCSD(T)	*D*_e_ CAM-B3LYP
Py^+^Bz(I)	141.4	–178.2	–17.0	–2.8	–56.6	–57.4	–62.4
Py^+^Tol(I)	153.9	–194.2	–18.4	–2.9	–61.6	–62.2	–67.9
Py^+^Bz(II)	433.5	–449.7	–26.9	–6.4	–49.5	–47.5	–52.6
Py^+^Tol(II)	452.4	–477.9	–30.4	–6.6	–62.6	–60.2	–66.4
Py_2_^+^	4324.0	–4366.0	–37.1	–13.6	–104.2	–93.0	–93.3

a See Table S5 for details. Dissociation energies (*D*_e_) are uncorrected for zero-point energies computed at the DLPNO-CCSD­(T)/def2-SVP and CAM-B3LYP-D3/aug-cc-pVTZ levels using the optimized geometries obtained at the CAM-B3LYP-D3/aug-cc-pVTZ level. While *D*_e_ takes monomer relaxation into account, *E*_int_ represents the bare intermolecular interaction between the monomers in the dimer configuration.

### 3.4 Correlation of Bound NH Stretch Frequency with Dissociation Energy of the NH···π H-Bond

It is long known that in H-bonded or proton-bound dimers, the redshift of the frequency of the proton donor stretch vibration is directly related to the strength of the H-bond (*D*_0_), which in turn is correlated with the difference in the proton affinity of the two bases.^90–92^ Such correlations have previously been demonstrated also for ν_NH_^b^ of H-bonded Py^(+)^L dimers.^62,93^ In general, this phenomenon also holds for neutral π H-bonds as well as cation-π H-bonds.^32,94–96^ The correlation between Δν_NH_^b^ and *D*_e_ for the NH···π H-bonds of Py^+^Bz and Py^+^Tol are visualized in Figure 6. The experimental Δν_NH_^b^ values for Py^+^, Py^+^Bz, and Py^+^Tol are 0, −264, and −297 cm^–1^, and their corresponding *D*_e_ values are computed as 0, 62.4, and 67.9 kJ mol^–1^, respectively. The systematic ν_NH_^b^ redshifts of Py^+^ are consistent with the NH···π H-bond strengths of Py^+^Bz and Py^+^Tol. Indeed, a roughly linear correlation is obtained between *D*_e_ and Δν_NH_^b^ for the cationic NH···π H-bonded clusters (*D*_e_ = 0.232 Δν_NH_^b^). The experimental Δν_NH_^b^ values for Py^+^L with L = Ar and N_2_ (−24 and −97 cm^–1^) and their corresponding *D*_e_ values (10.2 and 20.2 kJ mol^–1^, respectively, obtained at the CAM-B3LYP-D3 level) are compatible with the obtained linear correlation. Interestingly, the data point for L = H_2_O does not follow the linear correlation, probably because ν_NH_^b^ of Py^+^H_2_O is heavily perturbed by a FR typically observed for cationic NH···O H-bonds.^62,93^ In addition, the correlation could be different for π and σ H-bonds.^26^ The correlations between experimental/computed Δν_NH_^b^ and *D*_0_/*D*_e_ values for the NH···π H-bonds of Py^+^Bz and Py^+^Tol provided in Figure S9 show all the same trend as the one shown in Figure 6. We expand the correlation for the Py^+^Tol_2_ trimer, where Py^+^Tol is solvated by a second Tol ligand. The IRPD spectrum of Py^+^Tol_2_ recorded in the Tol loss channel is presented in Figure S10, and its isomer structures and their IR spectra computed at the CAM-B3LYP-D3/aug-cc-pVTZ level are summarized in Figures S11 and S12 (Table S6), respectively. The measured ν_NH_^b^ value is blueshifted by +19 cm^–1^ (3169 cm^–1^) from that of Py^+^Tol, indicating that the second Tol ligand reduces the NH···π H-bond strength of Py^+^ to the first Tol ligand. This noncoorperative three-body effect is typical for interior ion solvation and arises from increased charge delocalization, thereby reducing the electrostatic and induction attraction.^9^ The *D*_e_ value estimated from the correlation of 64.5 kJ mol^–1^ is comparable to the value computed for the Py^+^Tol_2_(I) cluster for loss of a single H-bonded Tol ligand (61.5 kJ mol^–1^, Figure S11a). The linear *D*_e_−Δν_NH_^b^ correlation holds well for Py^+^Tol_2_ (Figure 6) and therefore offers a valuable tool to estimate the dissociation energies of cationic NH···π H-bonds experimentally.

**Figure 6:**
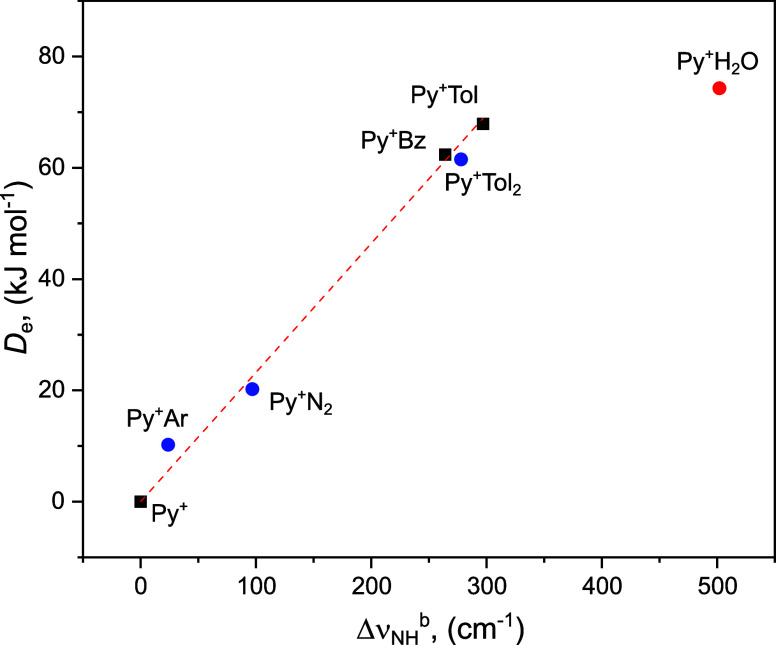
Correlation of observed Δν_NH_^b^ frequency shifts (absolute values) of Py^+^L with L = Ar, N_2_, Bz, Tol_*n*_ (*n* = 1 and 2), and H_2_O with computed dissociation energies (*D*_e_) of the corresponding H-bonds uncorrected for zero-point vibrational energy. The dashed line represents a linear fit to the data points of NH···π H-bonds.

## 4 Conclusions

Herein, we investigate the binding motifs of aromatic Py^+^X heterodimer radical cations as a function of ΔIE using IRPD spectroscopy of mass-selected bare and Ar-tagged Py^+^Bz and Py^+^Tol clusters. The spectroscopic efforts are complemented by DFT calculations at the CAM-B3LYP-D3/aug-cc-pVTZ level. Systematic shifts observed for the ν_NH_ stretch band of Py^+^ provide detailed information about the structures, binding motifs, and strengths of the corresponding intermolecular interactions in the observed cluster isomers. In both Py^+^Bz and Py^+^Tol, Py^+^ preferentially interacts with the π-ring of Bz or Tol via an ionic NH···π H-bond, favoring T-shaped structures over π-π stacking. This result is obtained because a strong CR, like in the Py_2_^+^ homodimer, cannot form efficiently between the π-systems of the two different aromatic molecules due to their large ΔIE values, thus allowing only for π-π stacking interactions stabilized by weaker electrostatic, induction, and dispersion forces, a view that is supported by the LED analysis of the individual contributions to the interaction. In general, the NH···π H-bond in Py^+^Tol is stronger than in Py^+^Bz, and this observation is attributed to the additional permanent electric dipole moment and the larger polarizability of Tol compared to Bz. The ν_NH_^b^ redshifts observed for Py^+^ upon dimer formation exhibit a linear correlation with the computed binding energy *D*_e_ (and *D*_0_) demonstrating that the measurement of ν_NH_^b^ can serve as an effective tool for evaluating the strength of the cationic NH···π H-bond. As an outlook, we are currently expanding these studies on aromatic heterodimer cations with lower ΔIE to locate the regime where the preferential interaction changes from π-π stacking or H-bond to CR.

## Supplementary Material



## Notes

Additional structures, measured and computed harmonic IR spectra, energies, LED data, and NCI plots of various isomers of Py^+^Bz­(Ar), Py^+^Tol­(Ar), and Py^+^Tol_2_ (PDF)
