# Supplementary material for: Competition between π‑π and NH···π Interactions in Pyrrole+‑Benzene and Pyrrole+‑Toluene Radical Cations Revealed by IR Spectroscopy
Source: J Phys Chem A. 2025 Oct 29;129(44):10161–71. doi: 10.1021/acs.jpca.5c05818 (PMC12598858; doi:10.1021/acs.jpca.5c05818)
Supplement: Supplementary file 1 [file jp5c05818_si_001.pdf]

Supporting Information

Competition Between π-π and NH…π Interactions in Pyrrole^{+}-Benzene and Pyrrole^{+}-Toluene Radical Cations Revealed by IR Spectroscopy

Dashjargal Arildii and Otto Dopfer*

Institut für Physik und Astronomie, Technische Universität Berlin, Hardenbergstrasse 36, 10623 Berlin, Germany

* Corresponding author: dopfer@physik.tu-berlin.de

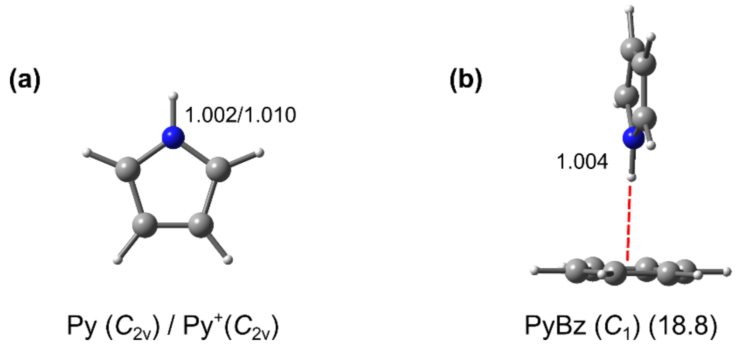

Figure S1. Structures of (a) Py$^{(+)}$ and (b) PyBz obtained at the CAM-B3LYP-D3/aug-cc-pVTZ level. Selected intramolecular bond lengths (in Å) are given in black. The dissociation energy of PyBz (D_0, in parentheses) is given in kJ mol^{-1}. The structures of Py and Py$^+$ in their $^1A_1$ and $^2A_2$ electronic ground states are planar (C_{2v}), and the N-H bond length increases by 8 mÅ from r_{NH}=1.002 to 1.010 Å upon ionization from the $\pi(a_2)$ HOMO orbital. This geometry change results in a large calculated redshift from 3526 to 3447 cm^{-1} ($\Delta\nu_{NH}^{f}$=-79 cm^{-1}), which compares favourably with the measured values of 3531 and 3447 cm^{-1} ($\Delta\nu_{NH}^{f}$=-84 cm^{-1}), respectively.[1,2]

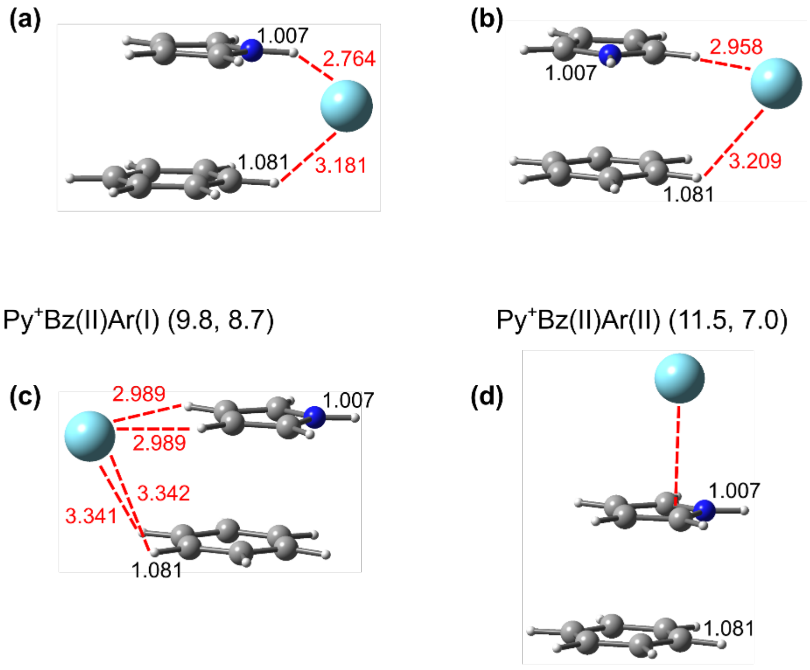

Py$^+$Bz(II)Ar(I) (9.8, 8.7)

Py$^+$Bz(II)Ar(II) (11.5, 7.0)

Py$^+$Bz(II)Ar(III) (12.0, 6.5)

Py$^+$Bz(II)Ar(IV) (12.9, 5.6)

Figure S2. Structures of (a-d) Py$^+$Bz(II)Ar(I-IV) obtained at the CAM-B3LYP-D3/aug-cc-pVTZ level. Selected intra- and intermolecular bond lengths (in Å) are given in black and red, respectively. Energies in parentheses are the relative energy and the dissociation energy of the most weakly bonded ligand (E_0 and D_0 in kJ mol^{-1}).

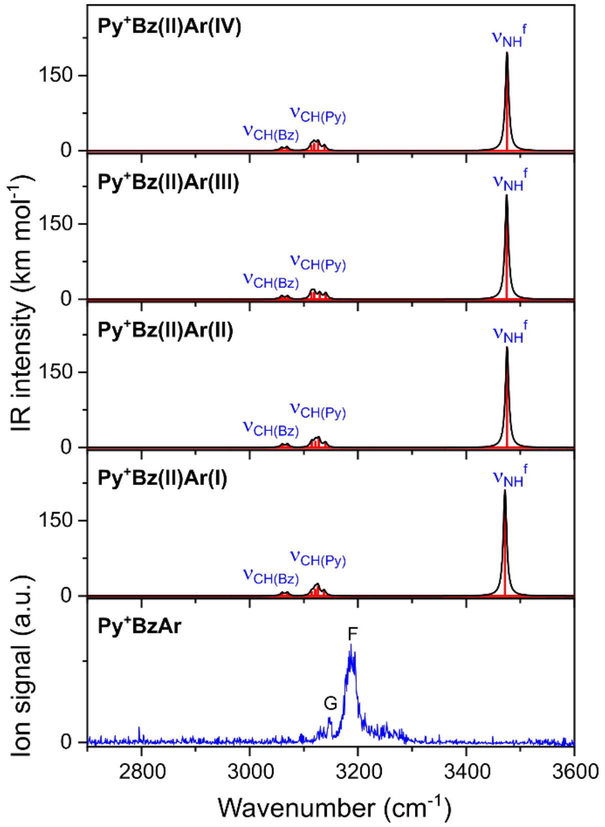

Figure S3. IRPD spectrum of Py^+BzAr recorded in the CH and NH stretch range compared to linear IR absorption spectra calculated for Py^+Bz(II)Ar(I-IV) at the CAM-B3LYP-D3/aug-cc-pVTZ level (Table S2).

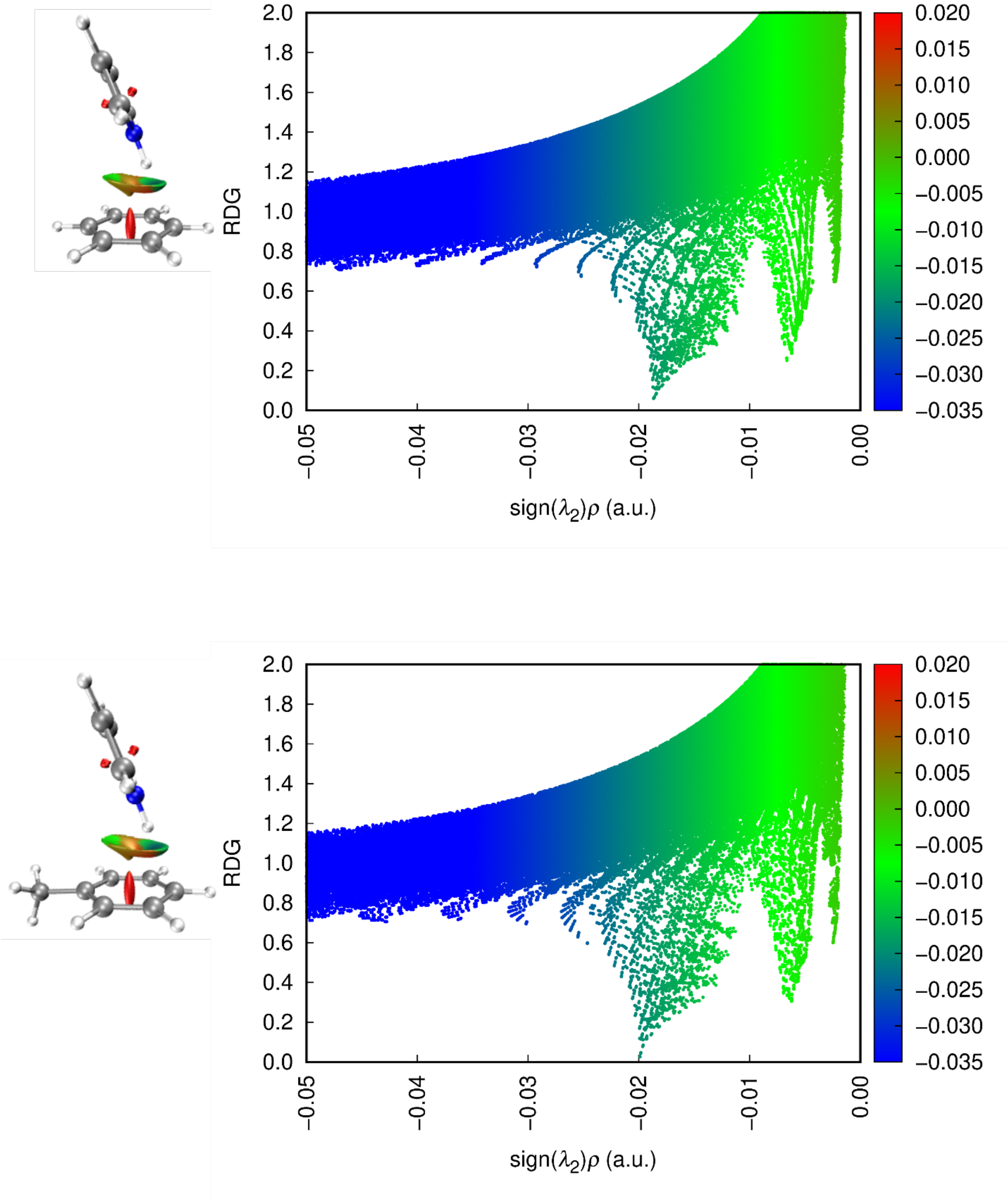

Figure S4. Noncovalent (NCI) interactions in T-shaped $Py^+Bz(I)$ (top) and $Py^+Tol(I)$ (bottom) visualized via the reduced electron density gradient (RDG), $s(\rho) \sim |\nabla(\rho)|/\rho^{4/3}$ as a function of the oriented electron density ρ^*=sign(λ_2)ρ. The NCI analysis is based on the evaluation of the electron density (ρ) and its reduced gradient $s(\rho)$.[3,4] It provides an index based on a plot of $s(\rho)$ in the area in which $s(\rho)$ is close to its minima of ρ. The final visualization is then obtained by plotting RDG against ρ oriented by the sign of the second eigenvalue λ_2 of the Hessian matrix, ρ^*=sign(λ_2)ρ. The plotted NCI surfaces cover the range $-0.05 < \rho^* < 0.0$ a.u., with an isosurface value of 0.3 a.u.. The NCI color code uses blue for attractive interactions (negative λ_2), green for weak van der Waals contacts ($\lambda_2 \leq 0$), and red for repulsive interactions (positive λ_2).

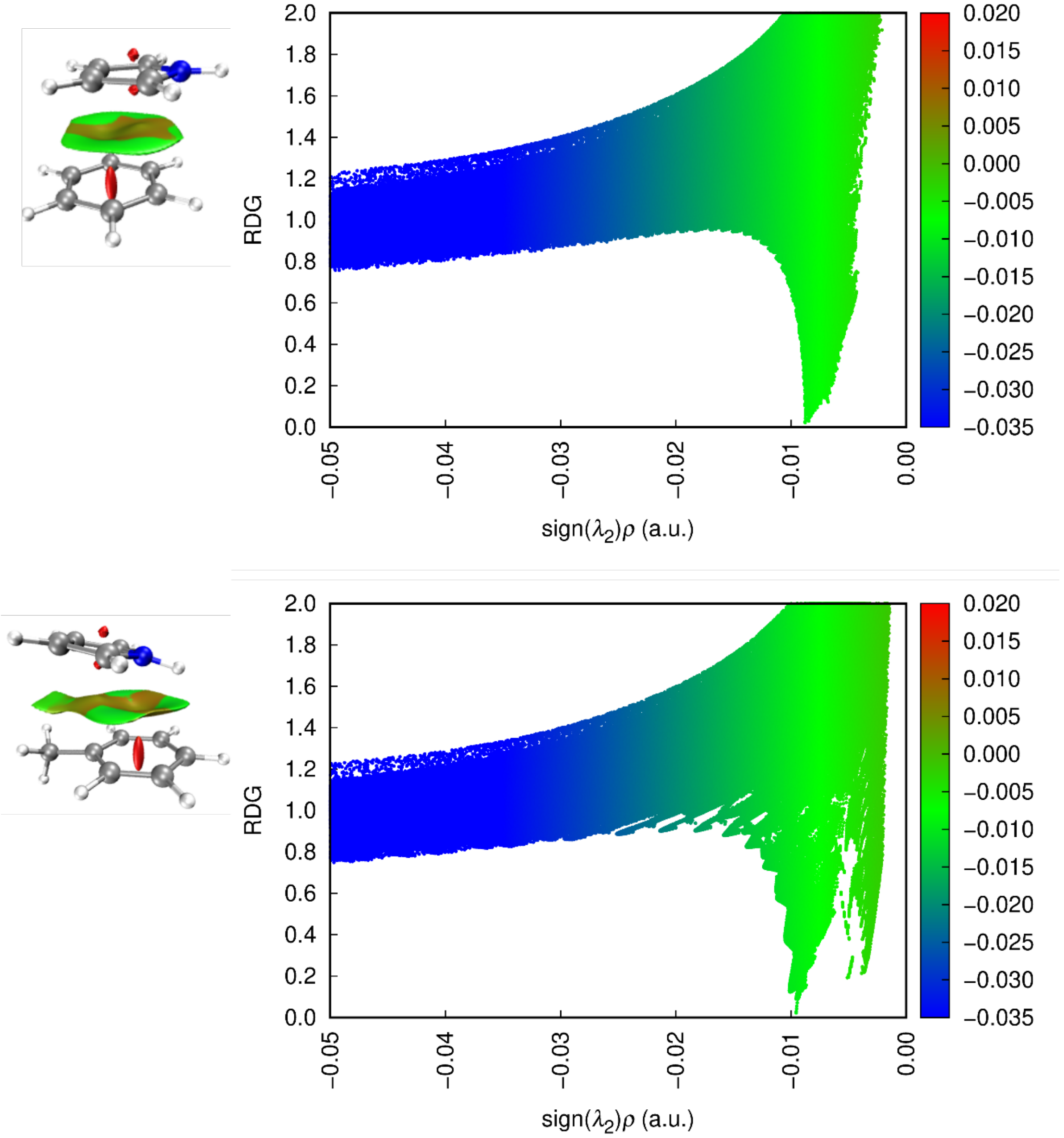

Figure S5. Noncovalent (NCI) interactions in π-stacked Py^+Bz(II) (top) and Py^+Tol(II) (bottom) visualized via the reduced electron density gradient (RDG), $s(\rho) \sim |\nabla(\rho)|/\rho^{4/3}$ as a function of the oriented electron density ρ^*=sign(λ_2)ρ. The plotted NCI surfaces cover the range -0.05 < ρ^* < 0.0 a.u., with an isosurface value of 0.3 a.u.. The NCI color code uses blue for attractive interactions (negative λ_2), green for weak van der Waals contacts ($\lambda_2 \leq 0$), and red for repulsive interactions (positive λ_2).

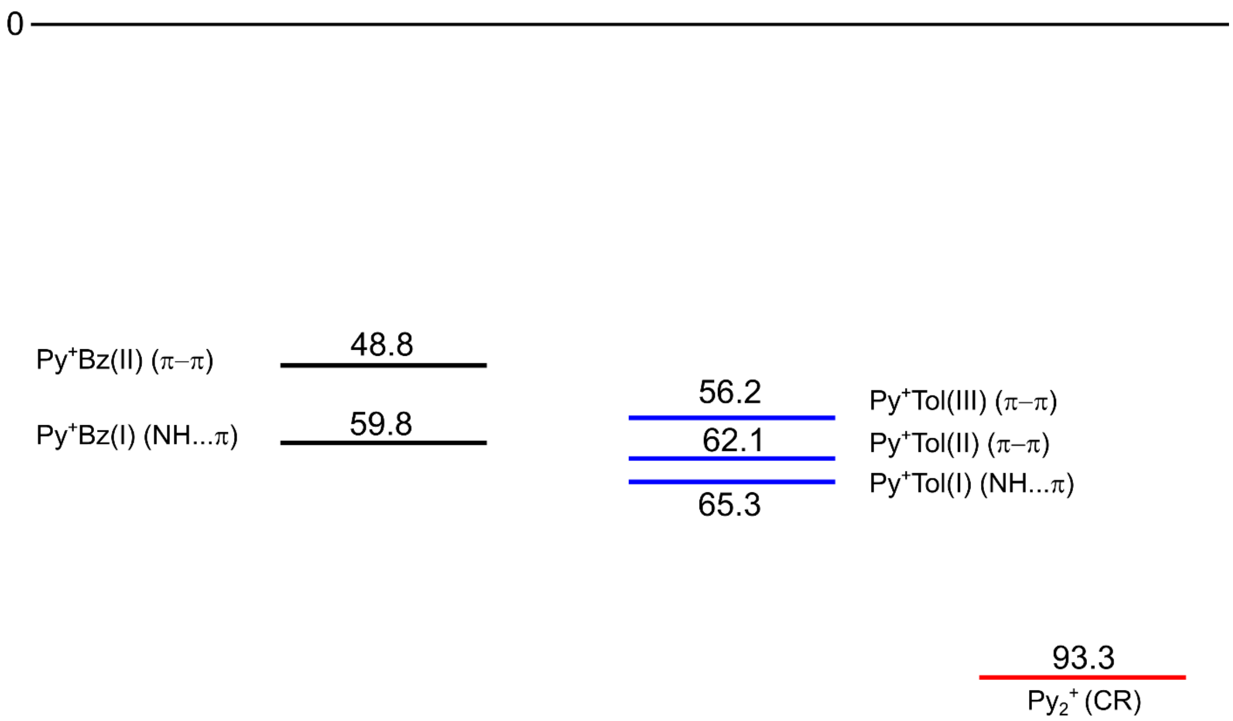

Figure S6. Direct comparison of the stabilisation energies of the $Py^{+}Bz$, $Py^{+}Tol$, and Py_2^{+} isomers obtained at the CAM-B3LYP-D3/aug-cc-pVTZ level (in kJ mol^{-1}).

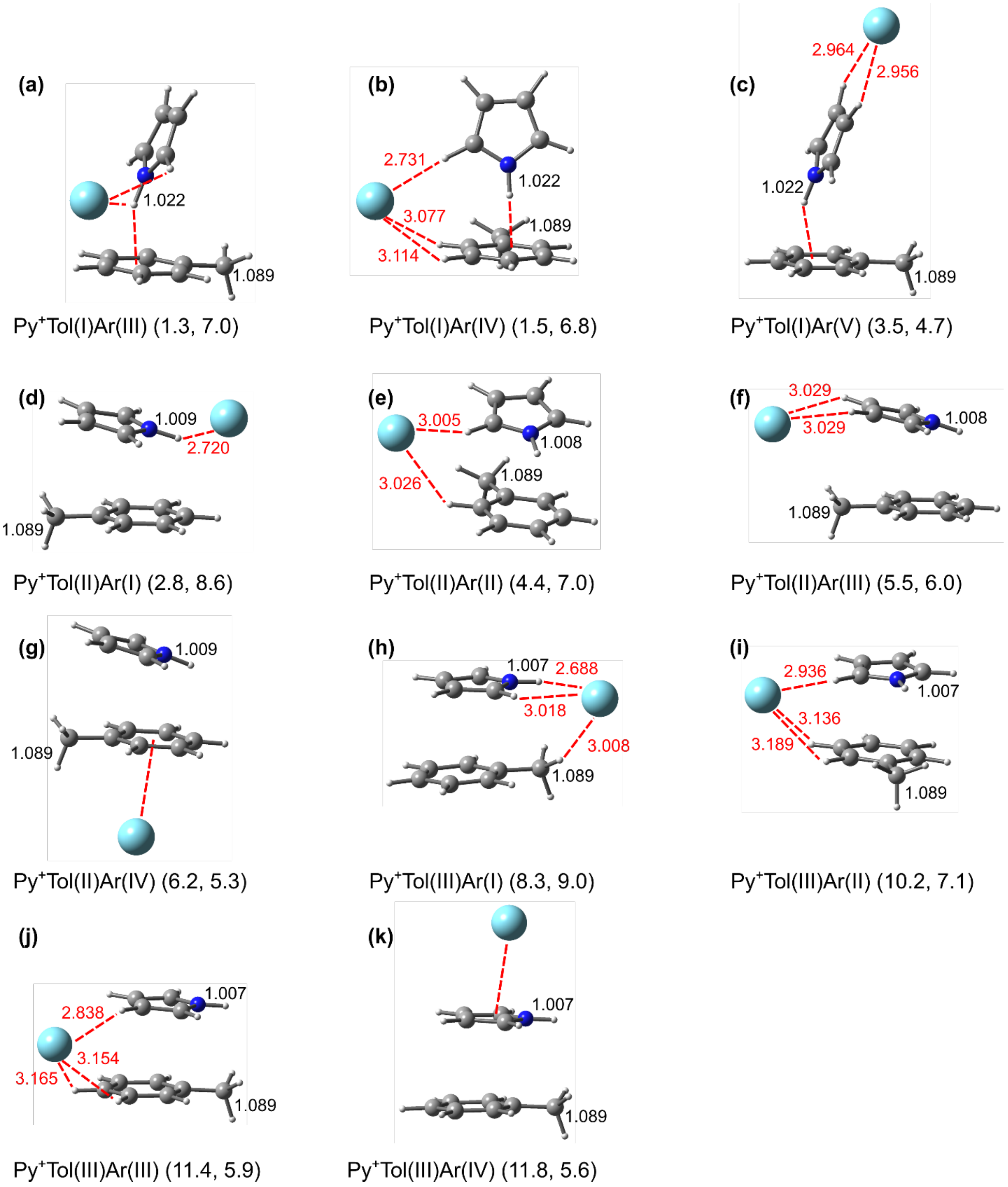

Figure S7. Structures of (a-c) $Py^+Tol(I)Ar(III\text{-}V)$, (d-g) $Py^+Tol(II)Ar(I\text{-}IV)$, and (h-k) $Py^+Tol(III)Ar(I\text{-}IV)$ obtained at the CAM-B3LYP-D3/aug-cc-pVTZ level. Selected intra- and intermolecular bond lengths (in Å) are given in black and red, respectively. Energies in parentheses are the relative energy and the dissociation energy of the most weakly bonded ligand (E_0 and D_0 in kJ mol^{-1}).

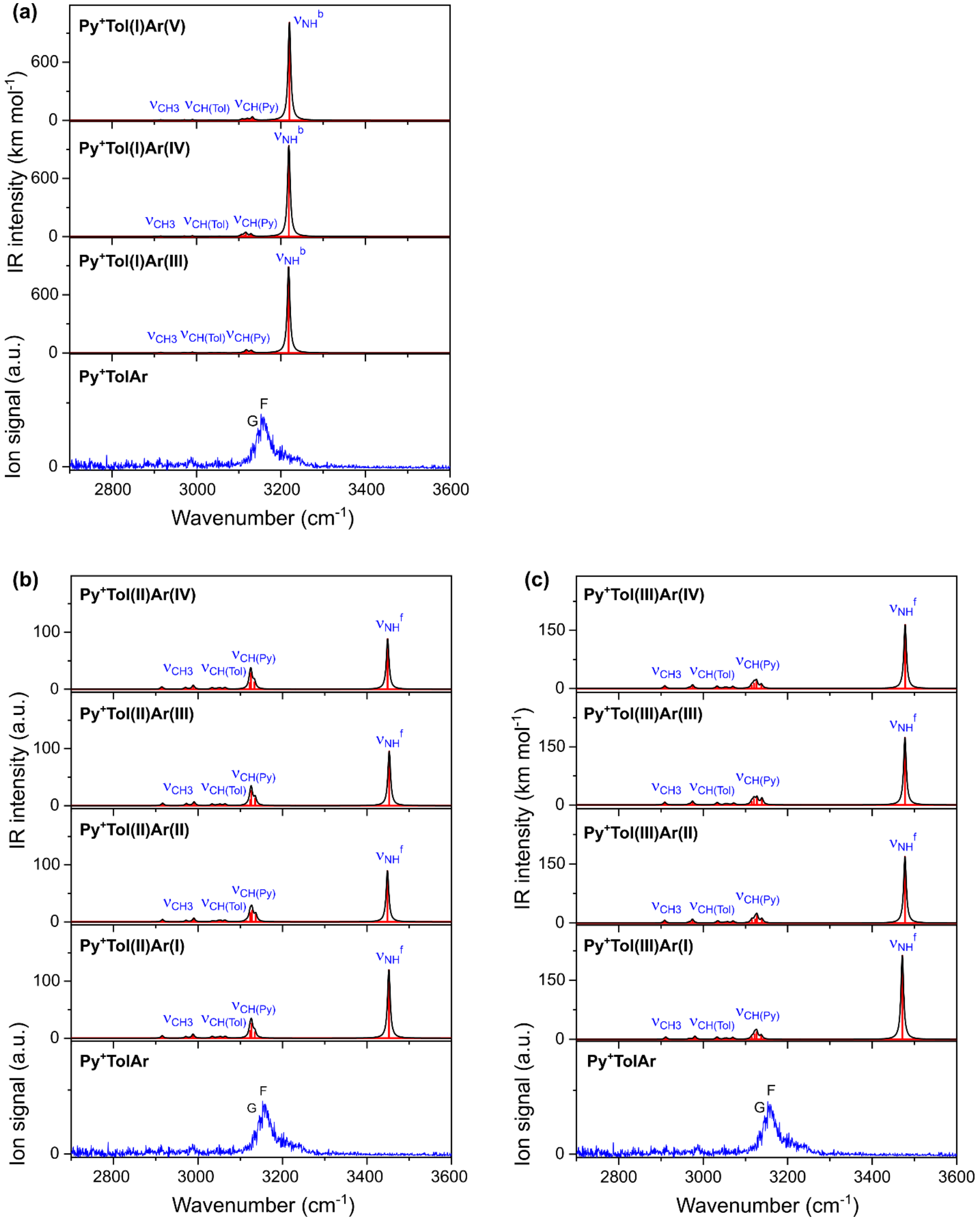

Figure S8. IRPD spectrum of Py^+TolAr recorded in the CH and NH stretch range compared to the linear IR absorption spectra calculated for (a) $Py^+Tol(I)(Ar)(III\text{-}V)$, (b) $Py^+Tol(II)(Ar)(I\text{-}IV)$, and (c) $Py^+Tol(III)(Ar)(I\text{-}IV)$, at the CAM-B3LYP-D3/aug-cc-pVTZ level (Tables S1 and S4).

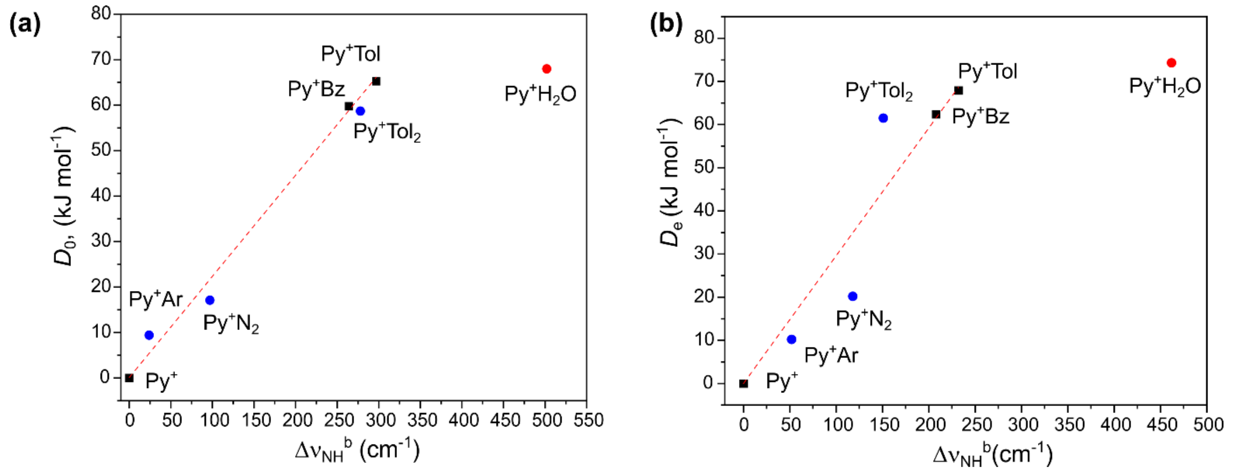

Figure S9. (a) Correlation of experimental $\Delta\nu_{NH}{}^{b}$ frequency shifts of $Py^{+}L$ with L=Ar, N_2, Bz, Tol_n (n=1 and 2), and H_2O with computed zero-point energy corrected dissociation energies (D_0) of the corresponding NH…π and NH…L H-bonds. (b) Correlation of computed $\Delta\nu_{NH}{}^{b}$ frequency shifts of $Py^{+}L$ with L=Ar, N_2, Bz, Tol_n (n=1 and 2), and H_2O with computed dissociation energies (D_e) for the H-bonds uncorrected for zero-point vibrational energy. The dashed lines represent a linear fit to the data points of the NH…π H-bonds.

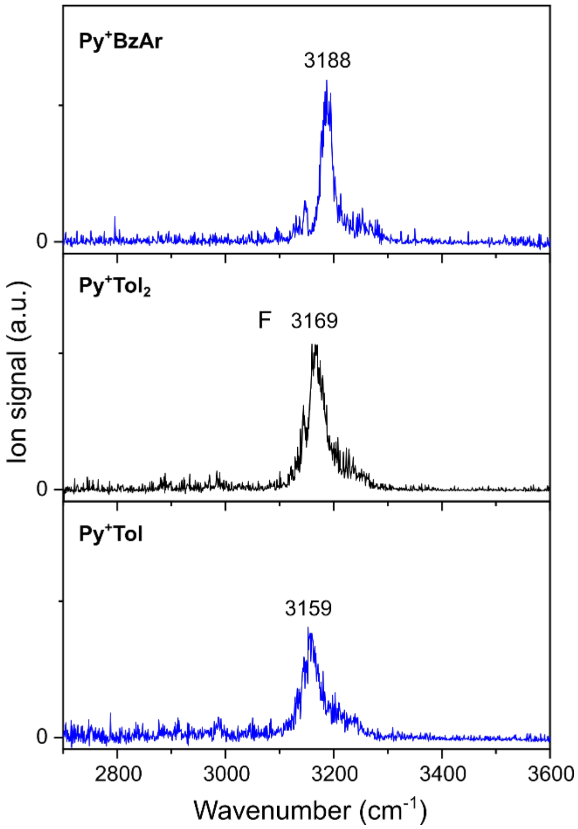

Figure S10. IRPD spectrum of $Py^{+}Tol_2$ recorded in the CH and NH stretch range compared to those of $Py^{+}BzAr$ and $Py^{+}TolAr$.

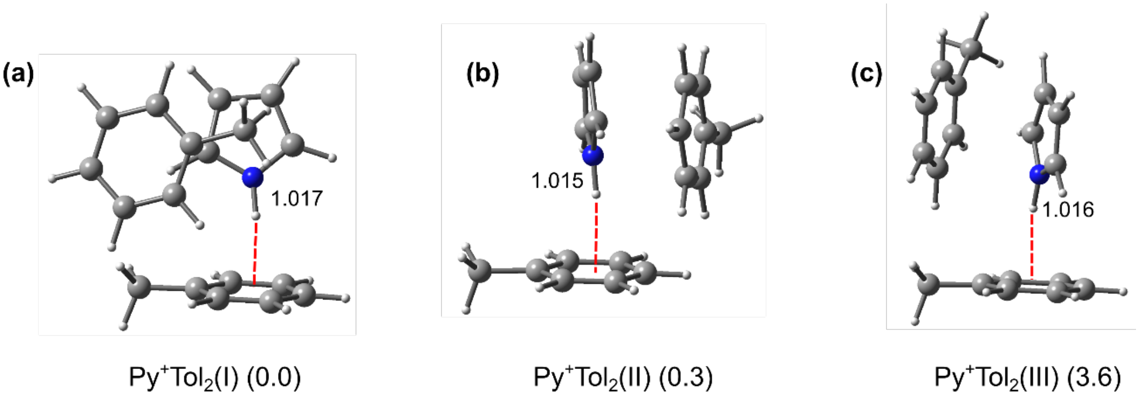

Figure S11. Structures of (a-c) $Py^{+}Tol_2$(I-III) obtained at the CAM-B3LYP-D3/aug-cc-pVTZ level. Selected intramolecular bond lengths (in Å) are given in black. Energies in parentheses are the relative energy (E_0) in kJ mol^{-1}.

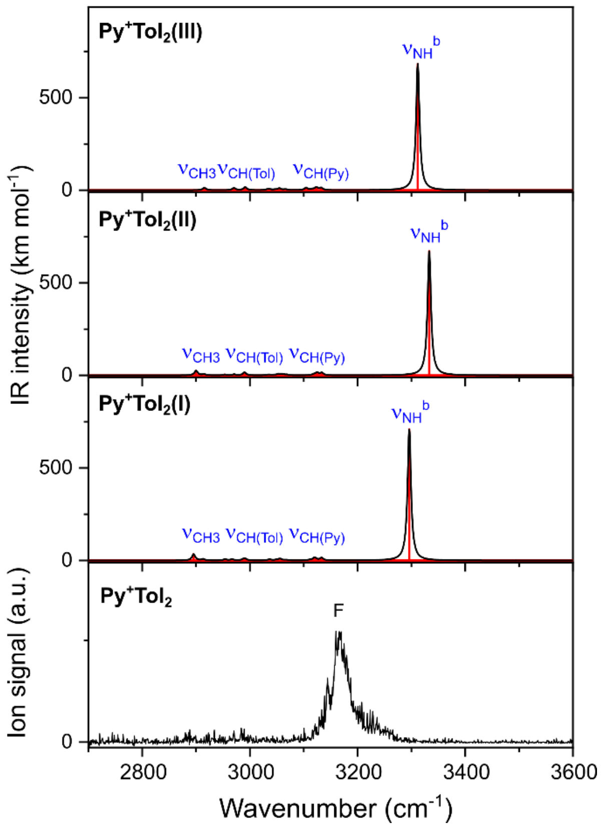

Figure S12. IRPD spectrum of $Py^{+}Tol_2$ recorded in the CH and NH stretch range compared to linear IR absorption spectra calculated for $Py^{+}Tol_2$(I-III) at the CAM-B3LYP-D3/aug-cc-pVTZ level (Table S5).

Table S1. Positions (in cm^{-1}) and suggested vibrational assignments of the transitions observed in the IRPD spectra of Py/Py^+,[1,2] PyBz,[5] Py^+Bz(Ar) and Py^+Tol(Ar) compared to frequencies of the most stable isomers calculated at the CAM-B3LYP-D3/aug-cc-pVTZ level.

	exp[a]	isomer	calc[b]	mode
Py	-		3098 (2), 3108 (2), 3122 (1), 3128 (0)	ν_{CH}
	3531		3523 (78)	ν_{NH}^{f}
Py^+	3131		3101 (20), 3107 (12), 3115 (29), 3127 (20)	ν_{CH}
	3447		3447 (228)	ν_{NH}^{f}
PyBz	3045, 3075, 3097		3035 (0), 3043 (0), 3045 (0), 3057 (15), 3059 (15), 3067 (0), 3096 (3), 3106 (3), 3121 (2), 3127 (0)	ν_{CH}
	3472		3511 (272)	ν_{NH}^{f}
Py^+Bz	Y 2915 (25)	Py^+Bz(I)	2934	FR/2β_{NH}
	G 3148 (nr)	Py^+Bz(I)	3039 (0.1), 3048 (0), 3059 (2), 3061 (2), 3068 (0.3), 3107 (7), 3113 (6), 3117 (23), 3129 (25)	ν_{CH}
	F 3183 (73)	Py^+Bz(I)	3239 (945)	ν_{NH}^{b}
	E 3470 (nr)	Py^+Bz(II)	3474 (202)	ν_{NH}^{f}
Py^+BzAr	G 3147 (12)	Py^+Bz(I)Ar(I)	3039 (0.1), 3045 (0), 3049 (0), 3059 (2), 3061 (2), 3068 (0.3) 3107 (7), 3113 (7), 3118 (23), 3129 (22)	ν_{CH}
	F 3188 (24)	Py^+Bz(I)Ar(I)	3238 (983), 3256 (898)	ν_{NH}^{b}
Py^+Tol	Y 2914 (13)	Py^+Tol(I)	2916 (5)	FR/2β_{NH} and ν_{CH3}
	G 3128 (nr)	Py^+Tol(I)	3034 (2), 3035 (1), 3047 (0.2), 3052 (3), 3065 (1), 3107 (7), 3113 (5), 3117 (23), 3129 (28)	$\nu_{CH(Py)}$
	F 3150 (63)	Py^+Tol(I)	3215 (982)	ν_{NH}^{b}
	E 3450 (nr)	Py^+Tol(II)	3449 (89)	ν_{NH}^{f}
Py^+TolAr	G 3132 (nr)	Py^+Tol(I)Ar(I)	3030 (2), 3038 (1), 3048 (1), 3054 (3), 3065 (1), 3107 (7), 3114 (6), 3118 (22), 3130 (24)	ν_{CH}
	F 3159 (38)	Py^+Tol(I)Ar(I)	3231 (948), 3214 (969)	ν_{NH}^{b}

[a] Peak positions and widths (in parentheses) are obtained from deconvolution. nr=not resolved, broad. [b]IR intensities in parenthesis are given in km mol^{-1}.

Table S2. Calculated NH and CH stretch frequencies of $Py^{+}Bz(II)$ and $Py^{+}Bz(II)Ar(I\text{-}IV)$ obtained at the CAM-B3LYP-D3/aug-cc-pVTZ level.

isomer	calc (IR intensity in km mol^{-1})	mode
$Py^{+}Bz(II)$	3036 (0.1), 3046 (0.3), 3048 (0.3), 3058 (4), 3060 (2), 3069 (7), 3113 (10), 3119 (14), 3126 (17), 3137 (10)	ν_{CH}
	3474 (202)	ν_{NH}^{f}
$Py^{+}Bz(II)Ar(I)$	3038 (0.2), 3046 (0.3), 3049 (0.3), 3060 (3), 3060 (4), 3069 (7), 3114 (8), 3121 (13), 3126 (18), 3138 (10)	ν_{CH}
	3472 (210)	ν_{NH}^{b}
$Py^{+}Bz(II)Ar(II)$	3037 (0.2), 3047 (0.3), 3049 (0.2), 3060 (4), 3061 (3), 3070 (7), 3115 (11), 3122 (12), 3128 (17), 3140 (10)	ν_{CH}
	3475 (200)	ν_{NH}^{f}
$Py^{+}Bz(II)Ar(III)$	3036 (0.1), 3046 (0.2), 3049 (0.5), 3059 (5), 3060 (2), 3070 (6), 3114 (13), 3119 (14), 3129 (12), 3141 (12)	ν_{CH}
	3475 (207)	ν_{NH}^{f}
$Py^{+}Bz(II)Ar(IV)$	3036 (0.1), 3046 (0.3), 3048 (0.3), 3059 (4), 3060 (2), 3069 (7), 3114 (10), 3119 (14), 3126 (17), 3138 (10)	ν_{CH}
	3475 (195)	ν_{NH}^{f}

Table S3. The computed relative and free energies for $Py^{+}Bz$ isomers at various computational levels.

	isomer	CAM-B3LYP-D3 [a]		PBE0-D3 [a]		M06-2X-D3 [a]		CBS-QB3	
$Py^{+}Bz$		E_0	G_0	E_0	G_0	E_0	G_0	E_0	G_0
	$Py^{+}Bz$(I)	0	0	0	0	0	0	0	0
	$Py^{+}Bz$(II)	10.9	20.0	3.3	10.3	6.6	13.0	10.8	12.4

[a]The basis set is aug-cc-pVTZ.

Table S4. Calculated NH and CH stretch frequencies of $Py^+Tol(I)Ar(III-V)$, $Py^+Tol(II)Ar(I-IV)$, $Py^+Tol(III)Ar(I-IV)$ obtained at the CAM-B3LYP-D3/aug-cc-pVTZ level.

isomer	calc (IR intensity in km mol^{-1})	mode
$Py^+Tol(I)Ar(III)$	2915 (5), 2971 (5), 2990 (7), 3032 (2), 3036 (2), 3047 (0.5), 3053 (3), 3065 (2), 3109 (3), 3116 (7), 3118 (25), 3130 (24)	ν_{CH}
	3218 (887)	$\nu_{NH}{}^{b}$
$Py^+Tol(I)Ar(IV)$	2915 (5), 2971 (5), 2990 (7), 3035 (2), 3037 (1), 3048 (0.3), 3054 (4), 3065 (1), 3107 (15), 3113 (11), 3117 (35), 3129 (24)	ν_{CH}
	3219 (938)	$\nu_{NH}{}^{b}$
$Py^+Tol(I)Ar(V)$	2915 (5), 2971 (5), 2991 (7), 3033 (2), 3035 (1), 3047 (0.3), 3052 (3), 3065 (1), 3108 (12), 3113 (4), 3120 (16), 3132 (33)	ν_{CH}
	3220 (1009)	$\nu_{NH}{}^{b}$
$Py^+Tol(II)Ar(I)$	2915 (4), 2971 (3), 2988 (7), 3033 (3), 3035 (1), 3047 (1), 3053 (2), 3065 (3), 3113 (0.1), 3123 (13), 3127 (25), 3134 (10)	ν_{CH}
	3452 (120)	$\nu_{NH}{}^{b}$
$Py^+Tol(II)Ar(II)$	2916 (4), 2972(3), 2990 (6), 3034 (2), 3038 (1), 3048 (2), 3053 (2), 3064 (3), 3114 (1), 3123 (15), 3128 (20), 3137 (12)	ν_{CH}
	3448 (89)	$\nu_{NH}{}^{f}$
$Py^+Tol(II)Ar(III)$	2916 (4), 2973 (4), 2991 (6), 3033 (2), 3035 (0.5), 3046 (1), 3052 (2), 3064 (3), 3124 (10), 3126 (26), 3135 (13)	ν_{CH}
	3453 (95)	$\nu_{NH}{}^{f}$
$Py^+Tol(II)Ar(IV)$	2914 (4), 2970 (3), 2989 (7), 3033 (2), 3035 (0.4), 3046 (1), 3052 (2), 3064 (3), 3113 (0), 3122 (11), 3125 (28), 3134 (12)	ν_{CH}
	3449 (88)	$\nu_{NH}{}^{f}$
$Py^+Tol(III)Ar(I)$	2911 (6), 2967 (2), 2981 (8), 3033 (4), 3034 (2), 3050 (2), 3056 (2), 3070 (5), 3115 (6), 3122 (13), 3126 (19), 3137 (9)	ν_{CH}
	3471 (213)	$\nu_{NH}{}^{b}$
$Py^+Tol(III)Ar(II)$	2909 (7), 2966 (2), 2975 (10), 3033 (3), 3036 (3), 3051 (2), 3058 (3), 3071 (5), 3115 (9), 3123 (9), 3127 (19), 3139 (10)	ν_{CH}
	3478 (168)	$\nu_{NH}{}^{f}$
$Py^+Tol(III)Ar(III)$	2909 (7), 2966 (2), 2975 (9), 3033 (5), 3034 (1), 3052 (2), 3058 (2), 3072 (5), 3115 (8), 3120 (14), 3127 (17), 3139 (16)	ν_{CH}
	3478 (174)	$\nu_{NH}{}^{f}$
$Py^+Tol(III)Ar(IV)$	2909 (7), 2966 (2), 2975 (9), 3033 (5), 3033 (1), 3051 (2), 3056 (2), 3070 (5), 3115 (7), 3120 (13), 3126 (17), 3138 (10)	ν_{CH}
	3478 (164)	$\nu_{NH}{}^{f}$

Table S5. Summary of the contributions of the LED analysis obtained at the DLPNO-CCSD(T)/def2-SVP level (in kJ mol^{-1}).

	$\Delta E^{ref.}_{el-prep.}$	$\Delta E^{ref.}_{elstat.}$	$\Delta E^{ref.}_{exch.}$	$\Delta E^{C-CCSD}_{non-disp.}$	$\Delta E^{C-CCSD}_{disp.}$	$\Delta E^{C-(T)}_{int}$	$\Delta E_{int.}$	D_e CCSD(T)	D_e CAM-B3LYP
$Py^+Bz(I)$	173.3	-177.3	-31.8	-1.0	-17.0	-2.8	-56.6	-57.4	-62.4
$Py^+Tol(I)$	188.2	-193.4	-34.4	-0.7	-18.4	-2.9	-61.6	-62.2	-67.9
$Py^+Bz(II)$	600.1	-438.9	-166.6	-10.8	-26.9	-6.4	-49.5	-47.5	-52.6
$Py^+Tol(II)$	617.5	-469.7	-165.2	-8.2	-30.4	-6.6	-62.6	-60.2	-66.4
Py_2^+	5637.6	-4425.6	-1248.3	-17.2	-37.1	-13.6	-104.2	-93.0	-93.3

To obtain the energies reported in Table 2, we used the following equations as recommended in refs. 6 and 7.

$E_{rep} = \Delta E^{ref.}_{el-prep.} + \Delta E^{ref.}_{exch.}$

$E_{es/ind} = \Delta E^{ref.}_{elstat.} + \Delta E^{C-CCSD}_{non-disp.}$

$E_{disp} = \Delta E^{C-CCSD}_{disp.}$

$E_{T} = \Delta E^{C-(T)}_{int}$

$E_{int} = \Delta E_{int.} = \Delta E^{ref.}_{el-prep.} + \Delta E^{ref.}_{exch.} + \Delta E^{ref.}_{elstat.} + \Delta E^{C-CCSD}_{non-disp.} + \Delta E^{C-CCSD}_{disp.} + \Delta E^{C-(T)}_{int}$

Table S6. Calculated NH and CH stretch frequencies of $Py^{+}Tol_2$(I-III) obtained at the CAM-B3LYP-D3/aug-cc-pVTZ level.

	calc (IR intensity in km mol^{-1})	mode
$Py^{+}Tol_2$(I)	2896 (34), 2913 (7), 2954 (7), 2967 (6), 2988 (6), 2992 (8), 3035 (2), 3038 (1), 3048 (1), 3050 (1), 3055 (4), 3057 (5), 3065 (1), 3068 (2), 3111 (3), 3119 (9), 3122 (8), 3133 (14)	ν_{CH}
	3296 (708)	ν_{NH}[b]
$Py^{+}Tol_2$(II)	2900 (25), 2915 (6), 2953 (4), 2971 (5), 2990 (9), 3031 (2), 3035 (1), 3036 (2), 3044 (1), 3045 (1), 3052 (4), 3053 (2), 3059 (5), 3063 (3), 3069 (3), 3113 (0.5), 3119 (4), 3125 (14), 3134 (13)	ν_{CH}
	3333 (670)	ν_{NH}[b]
$Py^{+}Tol_2$(III)	2915 (7), 2916 (7), 2970 (7), 2971 (5), 2991 (8), 3033 (3), 3036 (3), 3038 (1), 3046 (2), 3048 (1), 3053 (3), 3055 (7), 3066 (2), 3066 (3), 3105 (13), 3116 (5), 3124 (14), 3133 (12)	ν_{CH}
	3312 (683)	ν_{NH}[b]

References

(1) Schütz, M.; Matsumoto, Y.; Bouchet, A.; Öztürk, M.; Dopfer, O. Microsolvation of the Pyrrole Cation (Py^+) with Nonpolar and Polar Ligands: Infrared Spectra of Py^+-L_n with L=Ar, N_2, and H_2O ($n \leq 3$). *Phys. Chem. Chem. Phys.* **2017**, *19* (5), 3970–3986.

(2) Matsumoto, Y.; Honma, K. NH Stretching Vibrations of Pyrrole Clusters Studied by Infrared Cavity Ringdown Spectroscopy. *J. Chem. Phys.* **2007**, *127* (18), 184310.

(3) Lu, T.; Chen, F. Multiwfn: A Multifunctional Wavefunction Analyzer. *J. Comput. Chem.* **2012**, *33* (5), 580–592.

(4) Humphrey, W.; Dalke, A.; Schulten, K. VMD: Visual Molecular Dynamics. *J. Mol. Graph.* **1996**, *14* (1), 33–38.

(5) Pfaffen, C.; Infanger, D.; Ottiger, P.; Frey, H.-M.; Leutwyler, S. N–H...π Hydrogen -Bonding and Large-Amplitude Tipping Vibrations in Jet-Cooled Pyrrole – Benzene. *Phys. Chem. Chem. Phys.* **2011**, *13* (31), 14110–14118.

(6) Schneider, W. B.; Bistoni, G.; Sparta, M.; Saitow, M.; Riplinger, C.; Auer, A. A.; Neese, F. Decomposition of Intermolecular Interaction Energies within the Local Pair Natural Orbital Coupled Cluster Framework. *J. Chem. Theory Comput.* **2016**, *12* (10), 4778–4792.

(7) Altun, A.; Izsák, R.; Bistoni, G. Local Energy Decomposition of Coupled-Cluster Interaction Energies: Interpretation, Benchmarks, and Comparison with Symmetry-Adapted Perturbation Theory. *Int. J. Quantum Chem.* **2021**, *121* (3), e26339.

Cartesian coordinates

	Atomic Number	Coordinates (Angstroms)		
**Ar**				
1	18	0	0	0

Sum of electronic and zero-point Energies= -527.564057

Sum of electronic and thermal Energies= -527.562641

Sum of electronic and thermal Enthalpies= -527.561697

Sum of electronic and thermal Free Energies= -527.579269

N_2

1	7	0	0	0.54328
2	7	0	0	-0.54328

Sum of electronic and zero-point Energies= -109.521061

Sum of electronic and thermal Energies= -109.518700

Sum of electronic and thermal Enthalpies= -109.517756

Sum of electronic and thermal Free Energies= -109.539478

H_2O

1	8	0	0	0.1162
2	1	0	0.76431	-0.4648
3	1	0	-0.76431	-0.4648

Sum of electronic and zero-point Energies= -76.416663

Sum of electronic and thermal Energies= -76.413828

Sum of electronic and thermal Enthalpies= -76.412884

Sum of electronic and thermal Free Energies= -76.434298

Py

1	6	0	1.11685	0.32853
2	6	0	0.7092	-0.97574
3	6	0	-0.7092	-0.97574
4	6	0	-1.11685	0.32853
5	7	0	0	1.11494
6	1	0	0	2.11707
7	1	0	2.10251	0.75804
8	1	0	1.35578	-1.83564
9	1	0	-1.35578	-1.83564
10	1	0	-2.10251	0.75804

Sum of electronic and zero-point Energies= -210.054113

Sum of electronic and thermal Energies= -210.050238

Sum of electronic and thermal Enthalpies= -210.049294

Sum of electronic and thermal Free Energies= -210.079795

Py^{+}

1	6	0	1.10113	0.35062
2	6	0	0.68085	-1.00977
3	6	0	-0.68085	-1.00977
4	6	0	-1.10113	0.35062
5	7	0	0	1.13511
6	1	0	0	2.14483
7	1	0	2.09622	0.76713
8	1	0	1.34448	-1.85751
9	1	0	-1.34448	-1.85751
10	1	0	-2.09622	0.76713

Sum of electronic and zero-point Energies= -209.756594

Sum of electronic and thermal Energies= -209.752659

Sum of electronic and thermal Enthalpies= -209.751715

Sum of electronic and thermal Free Energies= -209.782975

PyBz

1	6	2.19283	1.17615	-0.00142
2	6	3.44203	0.6179	0.00118
3	6	3.2733	-0.78987	0.00103
4	6	1.92761	-1.03738	-0.00163
5	7	1.28088	0.16258	-0.00311
6	1	0.28398	0.27956	-0.00503
7	1	1.88153	2.20535	-0.00217
8	1	4.373	1.15743	0.00297
9	1	4.05061	-1.53392	0.00267
10	1	1.38011	-1.96251	-0.0026
11	6	-2.1534	1.15346	0.72139
12	6	-2.1581	1.18489	-0.66496
13	6	-2.05178	0.00539	-1.38537
14	6	-1.94084	-1.20514	-0.71957
15	6	-1.93617	-1.2365	0.66613
16	6	-2.04239	-0.05742	1.38682
17	1	-2.23326	2.07338	1.28329
18	1	-2.24172	2.1293	-1.18408
19	1	-2.04881	0.03026	-2.46581

20	1	-1.85321	-2.12421	-1.28166
21	1	-1.84509	-2.18008	1.18545
22	1	-2.03194	-0.08149	2.46722

Sum of electronic and zero-point Energies= -442.155273

Sum of electronic and thermal Energies= -442.144799

Sum of electronic and thermal Enthalpies= -442.143855

Sum of electronic and thermal Free Energies= -442.198082

Py^+Ar

1	6	-1.17476	1.10005	2E-6
2	6	-2.53639	0.68071	-1.6E-5
3	6	-2.53647	-0.6806	2E-6
4	6	-1.17488	-1.10009	-8E-6
5	7	-0.39071	-6.3E-5	0
6	1	0.62164	-1.16E-4	3E-6
7	1	-0.75818	2.09503	-1E-6
8	1	-3.38383	1.3446	-2.5E-5
9	1	-3.38398	-1.3444	5E-6
10	1	-0.75841	-2.09512	-2E-6
11	18	3.05181	2E-6	8E-6

Sum of electronic and zero-point Energies= -737.324241

Sum of electronic and thermal Energies= -737.317763

Sum of electronic and thermal Enthalpies= -737.316819

Sum of electronic and thermal Free Energies= -737.359159

Py^+N_2

1	6	0.98185	1.09879	2.14E-4
2	6	2.34432	0.68043	-3.32E-4
3	6	2.34431	-0.68053	-3.1E-4
4	6	0.98182	-1.09883	1.99E-4
5	7	0.19674	1.3E-5	5.14E-4
6	1	-0.81937	-5E-5	8.94E-4
7	1	0.56618	2.09409	3.35E-4
8	1	3.19158	1.34449	-6.44E-4
9	1	3.19158	-1.34457	-6.15E-4
10	1	0.56599	-2.09407	3.18E-4
11	7	-3.97036	-5.23E-4	-3.99E-4
12	7	-2.88492	6.54E-4	4E-5

Sum of electronic and zero-point Energies= -319.284168

Sum of electronic and thermal Energies= -319.276492

Sum of electronic and thermal Enthalpies= -319.275548

Sum of electronic and thermal Free Energies= -319.319003

Py$^+$H$_2$O

1	6	-0.51365	1.09482	-1.2E-5
2	6	-1.8793	0.67967	1.8E-5
3	6	-1.87905	-0.68004	2.9E-5
4	6	-0.51323	-1.09467	-5.1E-5
5	7	0.27318	2.18E-4	-1.6E-5
6	1	1.30959	2.9E-4	-5.7E-5
7	1	-0.09737	2.08965	-4E-6
8	1	-2.72618	1.34391	4E-5
9	1	-2.7257	-1.34458	5.4E-5
10	1	-0.09656	-2.08934	-6.9E-5
11	8	2.99852	-4E-6	1.2E-5
12	1	3.57348	-2.37E-4	0.77188
13	1	3.57367	1.93E-4	-0.77173

Sum of electronic and zero-point Energies= -286.199130

Sum of electronic and thermal Energies= -286.191698

Sum of electronic and thermal Enthalpies= -286.190754

Sum of electronic and thermal Free Energies= -286.231600

Bz

1	6	0	1.38549	0
2	6	1.19987	0.69275	0
3	6	1.19987	-0.69275	0
4	6	0	-1.38549	0
5	6	-1.19987	-0.69275	0
6	6	-1.19987	0.69275	0
7	1	0	2.46662	0
8	1	2.13616	1.23331	0
9	1	2.13616	-1.23331	0
10	1	0	-2.46662	0
11	1	-2.13616	-1.23331	0
12	1	-2.13616	1.23331	0

Sum of electronic and zero-point Energies= -232.094018

Sum of electronic and thermal Energies= -232.089685

Sum of electronic and thermal Enthalpies= -232.088741

Sum of electronic and thermal Free Energies= -232.119092

Bz$^+$

1	6	0	0	1.37125
2	6	0	1.24073	0.67888
3	6	0	1.24073	-0.67888
4	6	0	0	-1.37125
5	6	0	-1.24073	-0.67888
6	6	0	-1.24073	0.67888
7	1	0	0	2.45356
8	1	0	2.16253	1.2418
9	1	0	2.16253	-1.2418
10	1	0	0	-2.45356
11	1	0	-2.16253	-1.2418
12	1	0	-2.16253	1.2418

Sum of electronic and zero-point Energies= -231.760109

Sum of electronic and thermal Energies= -231.754966

Sum of electronic and thermal Enthalpies= -231.754022

Sum of electronic and thermal Free Energies= -231.787512

Tol

1	6	1.18926	1.19689	0.00183
2	6	-0.19463	1.19162	-0.00871
3	6	-0.90588	-0.00269	-0.01132
4	6	-0.19153	-1.19354	-0.00867
5	6	1.19377	-1.19441	0.00176
6	6	1.88993	0.00183	0.00831
7	1	1.7231	2.13722	0.00206
8	1	-0.73378	2.13024	-0.0177
9	1	-0.7268	-2.13424	-0.0173
10	1	1.7299	-2.13341	0.002
11	1	2.97078	0.00395	0.01435
12	6	-2.40751	-9.2E-4	0.00918
13	1	-2.81181	-0.92635	-0.39721
14	1	-2.78114	0.10125	1.0293
15	1	-2.81071	0.82864	-0.56983

Sum of electronic and zero-point Energies= -271.374783

Sum of electronic and thermal Energies= -271.368632

Sum of electronic and thermal Enthalpies= -271.367688

Sum of electronic and thermal Free Energies= -271.405660

Tol$^+$

1	6	1.18926	1.19689	0.00183
2	6	-0.19463	1.19162	-0.00871
3	6	-0.90588	-0.00269	-0.01132
4	6	-0.19153	-1.19354	-0.00867
5	6	1.19377	-1.19441	0.00176
6	6	1.88993	0.00183	0.00831
7	1	1.7231	2.13722	0.00206
8	1	-0.73378	2.13024	-0.0177
9	1	-0.7268	-2.13424	-0.0173
10	1	1.7299	-2.13341	0.002
11	1	2.97078	0.00395	0.01435
12	6	-2.40751	-9.2E-4	0.00918
13	1	-2.81181	-0.92635	-0.39721
14	1	-2.78114	0.10125	1.0293
15	1	-2.81071	0.82864	-0.56983

Sum of electronic and zero-point Energies= -271.374783

Sum of electronic and thermal Energies= -271.368632

Sum of electronic and thermal Enthalpies= -271.367688

Sum of electronic and thermal Free Energies= -271.405660

Py$^+$Bz(I)

1	6	-0.19928	1.85715	1.09656
2	6	0.17731	3.16928	0.68025
3	6	0.17731	3.16928	-0.68025
4	6	-0.19928	1.85715	-1.09656
5	7	-0.4193	1.10757	0
6	1	-0.67661	0.1196	0
7	1	-0.30978	1.44943	2.08833
8	1	0.41016	3.98379	1.34411
9	1	0.41016	3.98379	-1.34411
10	1	-0.30978	1.44943	-2.08833
11	6	0.7779	-1.80614	1.20155
12	6	-0.60246	-1.95275	1.20323
13	6	-1.2946	-2.02423	0
14	6	-0.60246	-1.95275	-1.20323
15	6	0.7779	-1.80614	-1.20155

16	6	1.46661	-1.73155	0
17	1	1.31876	-1.77334	2.13696
18	1	-1.1382	-2.03633	2.13858
19	1	-2.36688	-2.16085	0
20	1	-1.1382	-2.03633	-2.13858
21	1	1.31876	-1.77334	-2.13696
22	1	2.543	-1.63469	0

Sum of electronic and zero-point Energies= -441.873372

Sum of electronic and thermal Energies= -441.863116

Sum of electronic and thermal Enthalpies= -441.862172

Sum of electronic and thermal Free Energies= -441.916577

$Py^{+}Bz$(II)

1	6	-1.87293	-1.09879	0.06713
2	6	-1.36346	-0.70019	-1.18806
3	6	-1.35303	0.66854	-1.20506
4	6	-1.85526	1.10597	0.0403
5	7	-2.17713	0.01492	0.77366
6	1	-2.55174	0.02934	1.70839
7	1	-2.04712	-2.08568	0.46168
8	1	-1.05444	-1.37221	-1.9683
9	1	-1.03517	1.31633	-2.00208
10	1	-2.01346	2.10495	0.41026
11	6	1.4236	-1.37343	0.37263
12	6	1.92802	-0.81743	-0.80322
13	6	1.98472	0.55243	-0.94736
14	6	1.5329	1.37572	0.08039
15	6	1.05063	0.82191	1.26264
16	6	1.00063	-0.55219	1.40955
17	1	1.40475	-2.44781	0.49258
18	1	2.2861	-1.4632	-1.59232
19	1	2.38765	0.98902	-1.84997
20	1	1.59541	2.45	-0.0244
21	1	0.75282	1.46851	2.07661
22	1	0.66028	-0.98897	2.33832

Sum of electronic and zero-point Energies= -441.869213

Sum of electronic and thermal Energies= -441.859126

Sum of electronic and thermal Enthalpies= -441.858182

Sum of electronic and thermal Free Energies= -441.908979

Py$^+$Bz(I)Ar(I)

1	6	-1.51483	1.10382	1.1688
2	6	-2.80896	1.23484	0.58068
3	6	-2.62666	1.32235	-0.76417
4	6	-1.22192	1.24633	-0.9999
5	7	-0.59557	1.1203	0.18615
6	1	0.41287	1.03381	0.31323
7	1	-1.23346	1.00044	2.20436
8	1	-3.7354	1.25416	1.12805
9	1	-3.37554	1.42758	-1.52987
10	1	-0.66873	1.27324	-1.92437
11	6	1.81228	-1.26107	0.6102
12	6	1.77226	-1.16607	-0.77249
13	6	2.16324	0.00759	-1.40035
14	6	2.59109	1.09085	-0.64465
15	6	2.62995	0.99702	0.74131
16	6	2.24236	-0.18136	1.36796
17	1	1.52358	-2.18169	1.09694
18	1	1.45285	-2.01353	-1.36224
19	1	2.15975	0.07042	-2.47989
20	1	2.91532	1.99917	-1.13287
21	1	2.98448	1.83189	1.32934
22	1	2.29702	-0.26273	2.4446
23	18	-1.71092	-2.20226	-0.0397

Sum of electronic and zero-point Energies= -969.440316

Sum of electronic and thermal Energies= -969.427535

Sum of electronic and thermal Enthalpies= -969.426591

Sum of electronic and thermal Free Energies= -969.487134

Py$^+$Bz(I)Ar(II)

1	6	2.87299	-1.10093	0.14435
2	6	4.16098	-0.66309	-0.28764
3	6	4.14093	0.69701	-0.28005
4	6	2.84077	1.09185	0.15667
5	7	2.11792	-0.01689	0.40371
6	1	1.14221	-0.03237	0.70391
7	1	2.48548	-2.0994	0.26675

8	1	4.97402	-1.31355	-0.56011
9	1	4.93447	1.37407	-0.54509
10	1	2.42347	2.0767	0.29059
11	6	-0.85832	-1.29177	-0.48882
12	6	-0.94397	-1.09472	0.88278
13	6	-0.97142	0.19566	1.39821
14	6	-0.9162	1.28647	0.53922
15	6	-0.82912	1.08627	-0.83102
16	6	-0.79916	-0.20179	-1.34402
17	1	-0.86325	-2.29502	-0.89149
18	1	-1.01589	-1.94272	1.54943
19	1	-1.06326	0.35	2.46411
20	1	-0.96839	2.28977	0.9387
21	1	-0.80904	1.93454	-1.50071
22	1	-0.7513	-0.35604	-2.4126
23	18	-4.30549	0.0057	-0.13709

Sum of electronic and zero-point Energies= -969.439121

Sum of electronic and thermal Energies= -969.426282

Sum of electronic and thermal Enthalpies= -969.425338

Sum of electronic and thermal Free Energies= -969.488201

$Py^{+}Bz(II)Ar(I)$

1	6	-0.10707	2.01215	0.97601
2	6	-1.14686	2.11756	0.0262
3	6	-0.63573	1.71903	-1.1792
4	6	0.71526	1.36572	-0.96434
5	7	1.00492	1.57688	0.33989
6	1	1.89171	1.37882	0.77463
7	1	-0.10605	2.23868	2.02888
8	1	-2.14459	2.45604	0.24067
9	1	-1.14046	1.67292	-2.1273
10	1	1.45805	1.0097	-1.65764
11	6	-1.43973	-0.88734	1.47196
12	6	-2.61931	-0.73548	0.74052
13	6	-2.63479	-1.00272	-0.61549
14	6	-1.46908	-1.40227	-1.24973
15	6	-0.29018	-1.55768	-0.51956
16	6	-0.28304	-1.31625	0.84304

17	1	-1.44617	-0.71642	2.53956
18	1	-3.52631	-0.43152	1.24378
19	1	-3.55236	-0.90705	-1.17784
20	1	-1.47744	-1.62347	-2.30787
21	1	0.60337	-1.91395	-1.0131
22	1	0.61954	-1.47415	1.41678
23	18	3.40274	-0.81134	0.02688

Sum of electronic and zero-point Energies= -969.436592

Sum of electronic and thermal Energies= -969.424002

Sum of electronic and thermal Enthalpies= -969.423058

Sum of electronic and thermal Free Energies= -969.481205

Py^{+}Bz(II)Ar(II)

1	6	-1.56825	2.09779	-0.186
2	6	-0.99513	1.47067	-1.31238
3	6	0.31998	1.23073	-1.01735
4	6	0.54974	1.7107	0.29138
5	7	-0.60398	2.24465	0.75402
6	1	-0.73403	2.65421	1.66474
7	1	-2.57292	2.45176	-0.02698
8	1	-1.52008	1.23988	-2.22176
9	1	1.06474	0.76834	-1.63915
10	1	1.45402	1.71261	0.87574
11	6	-2.29191	-0.9008	0.70089
12	6	-2.21083	-1.46047	-0.57479
13	6	-1.0067	-1.96249	-1.04125
14	6	0.12213	-1.8812	-0.24742
15	6	0.04395	-1.31759	1.02779
16	6	-1.16811	-0.84805	1.50633
17	1	-3.246	-0.55551	1.07445
18	1	-3.09879	-1.528	-1.18758
19	1	-0.95185	-2.4189	-2.01901
20	1	1.06466	-2.27389	-0.60164
21	1	0.92262	-1.29628	1.65727
22	1	-1.23812	-0.45508	2.51116
23	18	3.46191	-0.2693	-0.01381

Py^{+}Bz(II)Ar(III)

1	6	1.23468	1.72788	1.10191
2	6	-0.09161	1.48362	0.68377
3	6	-0.09154	1.48335	-0.68504
4	6	1.23468	1.72732	-1.10312
5	7	2.00358	1.88884	-5.71E-4
6	1	2.99492	2.06608	-5.96E-4
7	1	1.64271	1.8133	2.09494
8	1	-0.92864	1.33206	1.34077
9	1	-0.9285	1.33141	-1.34201
10	1	1.64291	1.81219	-2.09612
11	6	0.70007	-1.57457	1.3838
12	6	-0.48708	-1.80446	0.69146
13	6	-0.4887	-1.8069	-0.68674
14	6	0.69673	-1.57907	-1.38259
15	6	1.88624	-1.37421	-0.69237
16	6	1.88795	-1.37222	0.69012
17	1	0.70411	-1.59642	2.46476
18	1	-1.40106	-1.99	1.23707
19	1	-1.40393	-1.99438	-1.22958
20	1	0.69824	-1.60428	-2.46348
21	1	2.8109	-1.25012	-1.23898
22	1	2.81396	-1.2466	1.23409
23	18	-3.41995	0.36891	-2.27E-4

Sum of electronic and zero-point Energies= -969.435762

Sum of electronic and thermal Energies= -969.423163

Sum of electronic and thermal Enthalpies= -969.422218

Sum of electronic and thermal Free Energies= -969.480475

Py^{+}Bz(II)Ar(IV)

1	6	-0.86541	0.85102	0.82731
2	6	-0.56828	-0.519	0.99642
3	6	-0.56739	-1.08963	-0.24738
4	6	-0.86372	-0.06805	-1.17618
5	7	-1.05316	1.08388	-0.4922
6	1	-1.27853	1.97586	-0.90162
7	1	-0.97034	1.6319	1.56149
8	1	-0.3865	-0.99679	1.94211

9	1	-0.38524	-2.11814	-0.50165
10	1	-0.96787	-0.11547	-2.24699
11	6	2.43721	0.73035	1.1753
12	6	2.73212	-0.63295	1.18206
13	6	2.76389	-1.33617	-0.00345
14	6	2.49543	-0.68289	-1.20276
15	6	2.22217	0.68162	-1.21253
16	6	2.19839	1.38811	-0.02402
17	1	2.44142	1.28438	2.10375
18	1	2.95091	-1.13052	2.11613
19	1	3.00677	-2.38908	-0.00582
20	1	2.53925	-1.22957	-2.1345
21	1	2.06763	1.19186	-2.15328
22	1	2.02173	2.45485	-0.02766
23	18	-4.19853	-0.22672	0.10027

Sum of electronic and zero-point Energies= -969.435390

Sum of electronic and thermal Energies= -969.422696

Sum of electronic and thermal Enthalpies= -969.421752

Sum of electronic and thermal Free Energies= -969.482072

$Py^{+}Tol(I)$

1	6	0.1953	2.04666	1.09628
2	6	-0.38468	3.28293	0.68011
3	6	-0.38468	3.28293	-0.68011
4	6	0.1953	2.04666	-1.09628
5	7	0.53105	1.34153	0
6	1	0.94253	0.4058	0
7	1	0.36942	1.66124	2.0878
8	1	-0.7434	4.05037	1.34394
9	1	-0.7434	4.05037	-1.34394
10	1	0.36942	1.66124	-2.0878
11	6	1.15761	-1.63253	1.19883
12	6	-0.22783	-1.6971	1.1942
13	6	-0.94203	-1.7347	0
14	6	-0.22783	-1.6971	-1.1942
15	6	1.15761	-1.63253	-1.19883
16	6	1.85894	-1.59705	0
17	1	1.69403	-1.62953	2.13773

18	1	-0.7634	-1.74219	2.1335
19	1	-0.7634	-1.74219	-2.1335
20	1	1.69403	-1.62953	-2.13773
21	1	2.93916	-1.56767	0
22	6	-2.43632	-1.86408	0
23	1	-2.87905	-1.40838	-0.88348
24	1	-2.72243	-2.91638	0
25	1	-2.87905	-1.40838	0.88348

Sum of electronic and zero-point Energies= -481.156242

Sum of electronic and thermal Energies= -481.144228

Sum of electronic and thermal Enthalpies= -481.143284

Sum of electronic and thermal Free Energies= -481.200943

Py^{+}Tol(II)

1	6	-0.83895	-1.55892	1.1006
2	6	0.21831	-2.40803	0.68267
3	6	0.21831	-2.40803	-0.68267
4	6	-0.83895	-1.55892	-1.1006
5	7	-1.46793	-1.09836	0
6	1	-2.17718	-0.38118	0
7	1	-1.17228	-1.30446	2.09191
8	1	0.87765	-2.94285	1.34361
9	1	0.87765	-2.94285	-1.34361
10	1	-1.17228	-1.30446	-2.09191
11	6	-0.66906	1.79973	1.19469
12	6	0.57492	1.17275	1.19132
13	6	1.22247	0.86691	0
14	6	0.57492	1.17275	-1.19132
15	6	-0.66906	1.79973	-1.19469
16	6	-1.29488	2.11925	0
17	1	-1.13384	2.06026	2.1358
18	1	1.06579	0.96124	2.13239
19	1	1.06579	0.96124	-2.13239
20	1	-1.13384	2.06026	-2.1358
21	1	-2.24453	2.63522	0
22	6	2.59327	0.26065	0
23	1	2.76124	-0.35225	-0.88361
24	1	3.35229	1.04343	0

25	1	2.76124	-0.35225	0.88361

Sum of electronic and zero-point Energies= -481.155027

Sum of electronic and thermal Energies= -481.143232

Sum of electronic and thermal Enthalpies= -481.142288

Sum of electronic and thermal Free Energies= -481.196210

Py$^+$Tol(III)

1	6	-0.99093	-1.58542	1.10276
2	6	0.2814	-2.02903	0.68483
3	6	0.2814	-2.02903	-0.68483
4	6	-0.99093	-1.58542	-1.10276
5	7	-1.74045	-1.35101	0
6	1	-2.68436	-1.00071	0
7	1	-1.38865	-1.4605	2.09551
8	1	1.08282	-2.30966	1.34429
9	1	1.08282	-2.30966	-1.34429
10	1	-1.38865	-1.4605	-2.09551
11	6	1.62429	0.87121	-1.19163
12	6	0.2814	1.25478	-1.18955
13	6	-0.39919	1.47671	0
14	6	0.2814	1.25478	1.18955
15	6	1.62429	0.87121	1.19163
16	6	2.30197	0.68998	0
17	1	2.13726	0.73694	-2.13374
18	1	-0.21971	1.43091	-2.13267
19	1	-0.21971	1.43091	2.13267
20	1	2.13726	0.73694	2.13374
21	1	3.34563	0.41202	0
22	6	-1.81789	1.96462	0
23	1	-2.35889	1.63484	0.88642
24	1	-1.84705	3.05435	0
25	1	-2.35889	1.63484	-0.88642

Sum of electronic and zero-point Energies= -481.152781

Sum of electronic and thermal Energies= -481.141060

Sum of electronic and thermal Enthalpies= -481.140116

Sum of electronic and thermal Free Energies= -481.192996

Py$^+$Tol(I)Ar(I)

1	6	-1.92111	-1.25089	-0.89751

2	6	-3.14551	-1.06173	-0.18712
3	6	-2.82205	-0.89539	1.12321
4	6	-1.40151	-0.98446	1.21577
5	7	-0.90489	-1.20055	-0.0179
6	1	0.08623	-1.29901	-0.24482
7	1	-1.75044	-1.40781	-1.95037
8	1	-4.12362	-1.05495	-0.63603
9	1	-3.48503	-0.72691	1.95399
10	1	-0.75188	-0.9054	2.0719
11	6	1.96251	-0.57589	-1.57519
12	6	1.80378	0.66204	-0.97093
13	6	1.88489	0.80248	0.41073
14	6	2.11788	-0.33836	1.17518
15	6	2.2773	-1.58023	0.57918
16	6	2.19899	-1.70541	-0.80249
17	1	1.91956	-0.65771	-2.65247
18	1	1.63125	1.53656	-1.58369
19	1	2.20496	-0.24719	2.25049
20	1	2.48002	-2.44941	1.18939
21	1	2.34446	-2.66809	-1.27149
22	6	1.77898	2.15142	1.05771
23	1	1.30468	2.09306	2.036
24	1	2.7726	2.57512	1.20678
25	1	1.21602	2.84896	0.44211
26	18	-1.55108	2.19028	-0.52544

Sum of electronic and zero-point Energies= -1008.723452

Sum of electronic and thermal Energies= -1008.708995

Sum of electronic and thermal Enthalpies= -1008.708051

Sum of electronic and thermal Free Energies= -1008.772013

Py$^+$Tol(I)Ar(II)

1	6	-2.25904	-1.47112	1.09942
2	6	-2.36968	-2.82931	0.67386
3	6	-2.37091	-2.8198	-0.6863
4	6	-2.2611	-1.45582	-1.09301
5	7	-2.20181	-0.68472	0.00862
6	1	-2.09228	0.33164	0.01536
7	1	-2.21744	-1.05723	2.09387

8	1	-2.4387	-3.67821	1.33184
9	1	-2.44114	-3.65942	-1.35594
10	1	-2.22147	-1.02775	-2.08151
11	6	-1.23701	2.19491	1.20951
12	6	-0.00902	1.55051	1.18891
13	6	0.61311	1.22646	-0.0134
14	6	-0.03498	1.56019	-1.19926
15	6	-1.26306	2.20464	-1.18801
16	6	-1.87324	2.52334	0.01882
17	1	-1.69119	2.45964	2.15445
18	1	0.4861	1.31471	2.12186
19	1	0.43992	1.33228	-2.14463
20	1	-1.73763	2.47703	-2.12068
21	1	-2.82014	3.04388	0.03121
22	6	1.96648	0.58109	-0.03059
23	1	2.10985	-0.03172	-0.91819
24	1	2.7478	1.34168	-0.03429
25	1	2.12779	-0.04127	0.84716
26	18	5.09733	-0.97793	0.00664

Sum of electronic and zero-point Energies= -1008.721322

Sum of electronic and thermal Energies= -1008.706611

Sum of electronic and thermal Enthalpies= -1008.705667

Sum of electronic and thermal Free Energies= -1008.775296

Py^+Tol(I)Ar(III)

1	6	0.63036	1.92358	1.03065
2	6	1.41993	3.00065	0.52734
3	6	2.05091	2.54268	-0.58736
4	6	1.64632	1.18535	-0.76692
5	7	0.79983	0.85724	0.22629
6	1	0.33114	-0.04408	0.33775
7	1	-0.01743	1.8775	1.89051
8	1	1.4842	3.97871	0.97177
9	1	2.73067	3.07363	-1.23103
10	1	1.91918	0.47681	-1.53165
11	6	-1.53226	-0.98297	1.59739
12	6	-2.34671	-0.04282	0.9838
13	6	-2.45736	0.01831	-0.40253

14	6	-1.72066	-0.88687	-1.16086
15	6	-0.90312	-1.82901	-0.55557
16	6	-0.80218	-1.88028	0.8292
17	1	-1.48038	-1.02975	2.6765
18	1	-2.92738	0.639	1.59176
19	1	-1.80288	-0.86541	-2.23955
20	1	-0.35453	-2.53555	-1.16277
21	1	-0.1802	-2.62552	1.30387
22	6	-3.38373	0.99752	-1.06031
23	1	-3.01504	1.3045	-2.03713
24	1	-4.36395	0.5443	-1.21191
25	1	-3.52898	1.88662	-0.44993
26	18	2.77766	-2.05324	-0.17229

Sum of electronic and zero-point Energies= -1008.722948

Sum of electronic and thermal Energies= -1008.708417

Sum of electronic and thermal Enthalpies= -1008.707473

Sum of electronic and thermal Free Energies= -1008.772318

Py^+Tol(I)Ar(IV)

1	6	1.79182	0.79091	0.03464
2	6	2.63135	1.83207	0.53367
3	6	2.00989	3.01026	0.25818
4	6	0.78889	2.69042	-0.40886
5	7	0.70295	1.35241	-0.52424
6	1	-0.07733	0.83832	-0.93817
7	1	1.92154	-0.27913	0.05717
8	1	3.57471	1.67941	1.02867
9	1	2.34619	4.00729	0.48434
10	1	0.01418	3.33978	-0.78353
11	6	-1.07787	-1.31336	-1.06867
12	6	-1.18773	-1.26531	0.31299
13	6	-1.92058	-0.2631	0.94204
14	6	-2.53464	0.69986	0.14629
15	6	-2.43011	0.6594	-1.23577
16	6	-1.69821	-0.34851	-1.85183
17	1	-0.52035	-2.11245	-1.53708
18	1	-0.71339	-2.03105	0.91254

19	1	-3.1192	1.48058	0.61493
20	1	-2.93502	1.4035	-1.83623
21	1	-1.63186	-0.39462	-2.92944
22	6	-2.08512	-0.24819	2.43278
23	1	-2.19225	0.76525	2.81463
24	1	-2.98264	-0.79821	2.7176
25	1	-1.24298	-0.72027	2.93493
26	18	2.05064	-3.00622	-0.02464

Sum of electronic and zero-point Energies= -1008.722888

Sum of electronic and thermal Energies= -1008.708353

Sum of electronic and thermal Enthalpies= -1008.707409

Sum of electronic and thermal Free Energies= -1008.772278

Py^{+}Tol(I)Ar(V)

1	6	0.83866	-0.85578	1.06464
2	6	2.15556	-0.478	0.66339
3	6	2.11785	-0.27583	-0.68107
4	6	0.7784	-0.53038	-1.1029
5	7	0.04945	-0.8782	-0.02545
6	1	-0.95009	-1.09078	-0.03168
7	1	0.45415	-1.09436	2.04307
8	1	3.00072	-0.38055	1.32215
9	1	2.92656	0.01778	-1.3272
10	1	0.33703	-0.47634	-2.08473
11	6	-3.01418	-0.96229	1.12932
12	6	-2.79665	0.40486	1.21357
13	6	-2.66782	1.18428	0.0677
14	6	-2.75494	0.55267	-1.16982
15	6	-2.97227	-0.81395	-1.26331
16	6	-3.10058	-1.5806	-0.11189
17	1	-3.13603	-1.54516	2.0319
18	1	-2.74715	0.87871	2.18516
19	1	-2.67546	1.14378	-2.07294
20	1	-3.06146	-1.28063	-2.23466
21	1	-3.29063	-2.64213	-0.18117
22	6	-2.4914	2.67067	0.16332
23	1	-1.94552	3.06644	-0.69088
24	1	-3.46367	3.16411	0.18359

25	1	-1.96518	2.95489	1.07244
26	18	5.53415	0.41877	0.00697

Sum of electronic and zero-point Energies= -1008.722101

Sum of electronic and thermal Energies= -1008.707524

Sum of electronic and thermal Enthalpies= -1008.706579

Sum of electronic and thermal Free Energies= -1008.773437

$Py^+Tol(II)Ar(I)$

1	6	-0.54819	1.5074	1.39006
2	6	-1.36798	2.32842	0.57
3	6	-0.75824	2.41637	-0.64843
4	6	0.43275	1.65134	-0.57513
5	7	0.53563	1.15091	0.67397
6	1	1.23594	0.48799	0.96938
7	1	-0.67007	1.21576	2.41863
8	1	-2.29105	2.78472	0.88196
9	1	-1.09152	2.95776	-1.51636
10	1	1.1917	1.47243	-1.31714
11	6	-0.55367	-1.81787	1.3969
12	6	-1.71889	-1.23649	0.89753
13	6	-1.84379	-0.91859	-0.45016
14	6	-0.75643	-1.16164	-1.28074
15	6	0.41024	-1.73669	-0.78726
16	6	0.51433	-2.07236	0.55514
17	1	-0.4981	-2.09077	2.44188
18	1	-2.55835	-1.07509	1.5613
19	1	-0.83312	-0.93669	-2.33655
20	1	1.23035	-1.94374	-1.46084
21	1	1.40859	-2.54778	0.93221
22	6	-3.12342	-0.3646	-0.99981
23	1	-2.93998	0.3314	-1.81656
24	1	-3.74462	-1.16896	-1.39465
25	1	-3.70294	0.14633	-0.23342
26	18	3.63297	0.04103	-0.23646

Sum of electronic and zero-point Energies= -1008.722377

Sum of electronic and thermal Energies= -1008.708083

Sum of electronic and thermal Enthalpies= -1008.707139

Sum of electronic and thermal Free Energies= -1008.769429

Py$^+$Tol(II)Ar(II)

1	6	0.60888	1.21024	-0.98273
2	6	1.06574	1.85795	0.19615
3	6	-0.00268	2.51564	0.73409
4	6	-1.11279	2.27082	-0.11301
5	7	-0.69412	1.51245	-1.14816
6	1	-1.29975	1.08744	-1.83374
7	1	1.14913	0.60895	-1.69259
8	1	2.07456	1.81721	0.5669
9	1	-0.02856	3.1157	1.62676
10	1	-2.13165	2.61003	-0.03557
11	6	-1.14169	-1.68843	-1.24409
12	6	-0.24911	-1.58099	-0.17819
13	6	-0.63436	-1.00834	1.0297
14	6	-1.9285	-0.51218	1.13321
15	6	-2.82048	-0.6096	0.07217
16	6	-2.431	-1.20424	-1.12249
17	1	-0.82735	-2.1737	-2.15795
18	1	0.74724	-1.99101	-0.27648
19	1	-2.25774	-0.07606	2.06728
20	1	-3.83233	-0.24589	0.18849
21	1	-3.13637	-1.31138	-1.93436
22	6	0.30395	-0.96336	2.19789
23	1	0.14817	-0.07098	2.80159
24	1	0.1411	-1.82441	2.84659
25	1	1.34405	-0.98917	1.87908
26	18	3.49003	-0.71494	-0.3525

Sum of electronic and zero-point Energies= -1008.721758

Sum of electronic and thermal Energies= -1008.707492

Sum of electronic and thermal Enthalpies= -1008.706548

Sum of electronic and thermal Free Energies= -1008.768220

Py$^+$Tol(II)Ar(III)

1	6	-0.16464	1.74009	-1.10108
2	6	1.15551	1.43199	-0.68293
3	6	1.15556	1.43243	0.68219
4	6	-0.16458	1.74079	1.10022

5	7	-0.91577	1.95058	-4.7E-4
6	1	-1.91724	2.06871	-4.76E-4
7	1	-0.57059	1.84488	-2.0924
8	1	1.98376	1.23749	-1.34107
9	1	1.98384	1.23835	1.34041
10	1	-0.57046	1.84621	2.09151
11	6	-2.66851	-0.49854	-1.19391
12	6	-1.39912	-1.07245	-1.19117
13	6	-0.75367	-1.38398	-2.61E-4
14	6	-1.39786	-1.07225	1.19128
15	6	-2.66723	-0.49834	1.19526
16	6	-3.30966	-0.21232	9.79E-4
17	1	-3.1635	-0.3005	-2.13485
18	1	-0.92637	-1.32091	-2.13249
19	1	-0.92413	-1.32056	2.13214
20	1	-3.16123	-0.30012	2.13667
21	1	-4.3076	0.20257	0.00148
22	6	0.58069	-2.06668	-8.96E-4
23	1	1.16385	-1.80945	0.88114
24	1	0.45124	-3.14931	-3.04E-4
25	1	1.16257	-1.81021	-0.88403
26	18	4.05596	-0.51809	4.17E-4

Sum of electronic and zero-point Energies= -1008.721354

Sum of electronic and thermal Energies= -1008.706995

Sum of electronic and thermal Enthalpies= -1008.706051

Sum of electronic and thermal Free Energies= -1008.769275

Py^{+}Tol(II)Ar(IV)

1	6	2.55167	-0.41192	1.10177
2	6	3.05509	0.84829	0.68709
3	6	3.05844	0.85048	-0.67821
4	6	2.55708	-0.40825	-1.09934
5	7	2.29872	-1.1466	-5.64E-4
6	1	1.81969	-2.03422	-0.00319
7	1	2.40366	-0.80666	2.09209
8	1	3.37164	1.63432	1.35008
9	1	3.3782	1.63867	-1.33708
10	1	2.41349	-0.7997	-2.09164

11	6	-0.70614	-1.23245	1.19308
12	6	-0.46912	0.14005	1.1887
13	6	-0.36647	0.8476	-0.00293
14	6	-0.46741	0.13819	-1.19354
15	6	-0.70435	-1.23436	-1.19607
16	6	-0.82928	-1.925	-0.00105
17	1	-0.82189	-1.75199	2.13447
18	1	-0.41366	0.67249	2.12927
19	1	-0.41046	0.66909	-2.13488
20	1	-0.81859	-1.75539	-2.13682
21	1	-1.04801	-2.98337	-3.47E-4
22	6	-0.19895	2.33663	-0.00395
23	1	0.33536	2.6808	-0.8875
24	1	-1.17465	2.82377	-0.00517
25	1	0.33384	2.68217	0.87998
26	18	-3.90795	0.31449	0.0023

Sum of electronic and zero-point Energies= -1008.721089

Sum of electronic and thermal Energies= -1008.706697

Sum of electronic and thermal Enthalpies= -1008.705753

Sum of electronic and thermal Free Energies= -1008.769145

Py$^+$Tol(III)Ar(I)

1	6	-0.75741	1.39684	-0.68087
2	6	0.49198	2.04186	-0.80438
3	6	0.99282	2.20055	0.46003
4	6	0.04849	1.65284	1.35514
5	7	-1.00383	1.20051	0.635
6	1	-1.81559	0.73419	1.00658
7	1	-1.46669	1.10561	-1.43642
8	1	0.94137	2.34109	-1.73409
9	1	1.92451	2.65283	0.74868
10	1	0.06729	1.594	2.4301
11	6	2.90355	-0.42413	0.52087
12	6	1.76489	-0.95463	1.13046
13	6	0.69558	-1.41779	0.37462
14	6	0.77031	-1.2884	-1.00522
15	6	1.90532	-0.75523	-1.61826
16	6	2.98041	-0.3331	-0.85577

17	1	3.73376	-0.10211	1.13383
18	1	1.73878	-1.0527	2.20812
19	1	-0.043	-1.65089	-1.62064
20	1	1.94845	-0.69463	-2.69682
21	1	3.86708	0.06052	-1.33055
22	6	-0.4918	-2.06054	1.02872
23	1	-1.39419	-1.94396	0.43044
24	1	-0.32742	-3.13123	1.15085
25	1	-0.67452	-1.65409	2.02311
26	18	-3.84987	-0.38954	-0.3439

Sum of electronic and zero-point Energies= -1008.720280

Sum of electronic and thermal Energies= -1008.706056

Sum of electronic and thermal Enthalpies= -1008.705112

Sum of electronic and thermal Free Energies= -1008.766617

Py^+Tol(III)Ar(II)

1	6	-0.62871	1.65967	0.28013
2	6	-0.58461	1.35389	-1.09772
3	6	0.67902	1.64571	-1.53825
4	6	1.40471	2.13194	-0.43202
5	7	0.57873	2.15089	0.64183
6	1	0.83981	2.42717	1.57408
7	1	-1.44391	1.57846	0.97826
8	1	-1.40954	0.96279	-1.66503
9	1	1.07221	1.53602	-2.5329
10	1	2.42309	2.47614	-0.36905
11	6	1.97193	-1.33878	-1.21002
12	6	2.22767	-0.87356	0.0804
13	6	1.23893	-0.88238	1.05357
14	6	-0.02853	-1.32039	0.68966
15	6	-0.28702	-1.79181	-0.59962
16	6	0.71591	-1.81254	-1.54892
17	1	2.76946	-1.34751	-1.93984
18	1	3.22673	-0.54488	0.33609
19	1	-0.81818	-1.3458	1.42931
20	1	-1.27542	-2.15458	-0.84385
21	1	0.52371	-2.19208	-2.54165
22	6	1.53359	-0.45432	2.46091

23	1	0.65273	-0.04029	2.95077
24	1	1.85608	-1.30623	3.05977
25	1	2.33712	0.28047	2.49968
26	18	-3.57013	-0.29392	0.20794

Sum of electronic and zero-point Energies= -1008.719552

Sum of electronic and thermal Energies= -1008.705293

Sum of electronic and thermal Enthalpies= -1008.704349

Sum of electronic and thermal Free Energies= -1008.765733

Py^+Tol(III)Ar(III)

1	6	-0.59518	1.85892	-1.00604
2	6	0.50297	1.67489	-0.13882
3	6	0.01232	1.66427	1.13993
4	6	-1.3839	1.84189	1.0536
5	7	-1.71309	1.98058	-0.25285
6	1	-2.64729	2.0966	-0.60981
7	1	-0.6237	1.93542	-2.07964
8	1	1.52507	1.56295	-0.45259
9	1	0.56281	1.54472	2.05565
10	1	-2.12382	1.90194	1.83353
11	6	-0.27713	-1.53954	1.53343
12	6	-1.55144	-1.28237	1.02328
13	6	-1.76407	-1.15252	-0.34204
14	6	-0.66157	-1.2355	-1.18263
15	6	0.61436	-1.49128	-0.67603
16	6	0.80704	-1.65521	0.68291
17	1	-0.14698	-1.66684	2.59907
18	1	-2.39299	-1.2331	1.70222
19	1	-0.79849	-1.15057	-2.25301
20	1	1.44997	-1.57648	-1.35627
21	1	1.79083	-1.8674	1.0743
22	6	-3.1443	-0.9525	-0.89496
23	1	-3.12821	-0.39193	-1.82917
24	1	-3.61214	-1.91298	-1.11173
25	1	-3.79292	-0.43845	-0.18638
26	18	3.92083	0.05254	-0.26511

Sum of electronic and zero-point Energies= -1008.719093

Sum of electronic and thermal Energies= -1008.704758

Sum of electronic and thermal Enthalpies= -1008.703814

Sum of electronic and thermal Free Energies= -1008.766393

Py$^+$Tol(III)Ar(IV)

1	6	0.95107	0.22884	-1.10334
2	6	0.80546	-1.11073	-0.68464
3	6	0.80517	-1.10982	0.68451
4	6	0.95057	0.23026	1.10146
5	7	1.05755	1.00699	-0.00147
6	1	1.15518	2.00896	-0.00205
7	1	1.01193	0.64069	-2.09627
8	1	0.71596	-1.9554	-1.34368
9	1	0.71555	-1.95365	1.3446
10	1	1.01095	0.64352	2.09383
11	6	-2.3945	-1.08084	1.19203
12	6	-2.16602	0.29658	1.1899
13	6	-2.07449	1.0064	2.29E-4
14	6	-2.16628	0.29658	-1.18943
15	6	-2.39469	-1.08081	-1.19149
16	6	-2.52059	-1.77079	3.19E-4
17	1	-2.49293	-1.602	2.13407
18	1	-2.11011	0.825	2.13291
19	1	-2.11058	0.82502	-2.13245
20	1	-2.49328	-1.60202	-2.13348
21	1	-2.71619	-2.83299	3.16E-4
22	6	-1.90638	2.49723	2.14E-4
23	1	-1.37657	2.84421	-0.88636
24	1	-2.87805	2.99144	6.35E-4
25	1	-1.37593	2.8441	0.88647
26	18	4.34473	-0.06351	7.32E-4

Sum of electronic and zero-point Energies= -1008.718961

Sum of electronic and thermal Energies= -1008.704609

Sum of electronic and thermal Enthalpies= -1008.703665

Sum of electronic and thermal Free Energies= -1008.766483

Py$^+$Tol$_2$(I)

1	6	-0.53244	0.806	-1.01613
2	6	-1.21456	1.89532	-1.6313
3	6	-0.72163	3.04422	-1.08012

4	6	0.2663	2.66072	-0.14227
5	7	0.35046	1.31461	-0.13671
6	1	0.99261	0.76226	0.42611
7	1	-0.61485	-0.25278	-1.19103
8	1	-1.97603	1.79403	-2.38389
9	1	-1.00673	4.05803	-1.29987
10	1	0.89358	3.26511	0.4914
11	6	2.57731	-0.27951	1.69348
12	6	2.06942	-1.2834	0.88156
13	6	2.35544	-1.3188	-0.48146
14	6	3.15995	-0.31508	-1.00899
15	6	3.6715	0.6906	-0.20345
16	6	3.38243	0.71408	1.15169
17	1	2.35926	-0.28228	2.75248
18	1	1.45379	-2.06021	1.31467
19	1	3.39978	-0.32693	-2.06382
20	1	4.30735	1.45121	-0.63424
21	1	3.79538	1.48573	1.78576
22	6	1.83351	-2.43172	-1.34231
23	1	2.4248	-3.33604	-1.19665
24	1	0.80253	-2.67896	-1.09112
25	1	1.87982	-2.17757	-2.39912
26	6	-3.30311	-1.14504	-0.88276
27	6	-3.24982	-0.02695	-0.06819
28	6	-2.40157	0.01535	1.04378
29	6	-1.61735	-1.10384	1.31612
30	6	-1.66854	-2.22104	0.50304
31	6	-2.50766	-2.24409	-0.60313
32	1	-3.97589	-1.1644	-1.72833
33	1	-3.88814	0.82171	-0.27618
34	1	-0.97102	-1.09914	2.18345
35	1	-1.06444	-3.08597	0.73818
36	1	-2.55495	-3.1227	-1.23024
37	6	-2.36717	1.22567	1.92274
38	1	-1.67972	1.10036	2.75594
39	1	-2.0675	2.11277	1.35885
40	1	-3.35486	1.43867	2.33068

Sum of electronic and zero-point Energies= -752.551065

Sum of electronic and thermal Energies= -752.531455

Sum of electronic and thermal Enthalpies= -752.530511

Sum of electronic and thermal Free Energies= -752.604252

$Py^+Tol_2(II)$

1	6	-0.85127	1.13891	-0.94548
2	6	-1.34274	2.46099	-0.73933
3	6	-0.46938	3.09092	0.10265
4	6	0.55647	2.16652	0.39997
5	7	0.29646	1.01276	-0.25463
6	1	0.86508	0.17269	-0.21104
7	1	-1.24277	0.34439	-1.55725
8	1	-2.24204	2.85401	-1.17905
9	1	-0.52511	4.09703	0.47941
10	1	1.43026	2.26575	1.02065
11	6	1.86525	-1.96183	-0.03058
12	6	2.63465	-1.29824	-0.97494
13	6	3.45754	-0.23467	-0.6119
14	6	3.47762	0.151	0.72493
15	6	2.70993	-0.50793	1.67357
16	6	1.90025	-1.56874	1.29974
17	1	1.2483	-2.79616	-0.33376
18	1	2.60903	-1.62066	-2.00747
19	1	4.12156	0.96568	1.03054
20	1	2.75755	-0.20332	2.70995
21	1	1.31619	-2.09546	2.04088
22	6	4.32945	0.44359	-1.6265
23	1	4.55437	1.46938	-1.34151
24	1	5.27927	-0.08399	-1.71998
25	1	3.86584	0.45251	-2.61113
26	6	-2.42541	-2.06523	-0.79007
27	6	-1.87489	-1.58503	0.38353
28	6	-2.34193	-0.40673	0.96324
29	6	-3.39369	0.2682	0.33366
30	6	-3.94997	-0.21407	-0.8394
31	6	-3.46233	-1.37793	-1.40927
32	1	-2.05652	-2.98501	-1.22159

33	1	-1.07531	-2.13019	0.86531
34	1	-3.78782	1.16847	0.78655
35	1	-4.77145	0.31343	-1.30301
36	1	-3.89786	-1.76075	-2.32088
37	6	-1.76316	0.11682	2.23966
38	1	-0.87921	-0.44127	2.53934
39	1	-1.49246	1.17087	2.14623
40	1	-2.49038	0.05397	3.04936

Sum of electronic and zero-point Energies= -752.550970

Sum of electronic and thermal Energies= -752.531199

Sum of electronic and thermal Enthalpies= -752.530255

Sum of electronic and thermal Free Energies= -752.605100

$Py^{+}Tol_{2}$(III)

1	6	-1.07465	2.46747	-0.39095
2	6	0.01156	3.36275	-0.56043
3	6	1.12128	2.73699	-0.07644
4	6	0.71172	1.44845	0.38865
5	7	-0.61109	1.33483	0.18484
6	1	-1.17278	0.51892	0.41427
7	1	-2.11606	2.57521	-0.64221
8	1	-0.05908	4.34494	-0.99383
9	1	2.13094	3.10592	-0.03859
10	1	1.28939	0.66682	0.85341
11	6	-3.95119	0.50617	0.15861
12	6	-3.44688	-0.34564	-0.81134
13	6	-2.58168	-1.38374	-0.47553
14	6	-2.2225	-1.53101	0.86174
15	6	-2.72313	-0.6785	1.83681
16	6	-3.59111	0.34447	1.48823
17	1	-4.64458	1.28766	-0.12167
18	1	-3.7469	-0.21966	-1.84349
19	1	-1.5604	-2.33694	1.14841
20	1	-2.44354	-0.82306	2.87093
21	1	-3.99449	0.99922	2.24716
22	6	-2.09714	-2.34507	-1.52025
23	1	-1.18944	-2.85696	-1.20815
24	1	-2.85185	-3.10927	-1.70793

25	1	-1.90493	-1.84429	-2.4678
26	6	2.42433	-0.13885	-1.58886
27	6	3.48653	0.08498	-0.7239
28	6	3.60232	-0.6222	0.46652
29	6	2.61911	-1.56378	0.7695
30	6	1.56308	-1.80011	-0.09489
31	6	1.45779	-1.08637	-1.27824
32	1	2.36446	0.40977	-2.51865
33	1	4.24575	0.8099	-0.98677
34	1	2.6964	-2.13157	1.68733
35	1	0.827	-2.55125	0.15381
36	1	0.64203	-1.2765	-1.96077
37	6	4.77479	-0.4197	1.37998
38	1	4.48833	-0.51692	2.42556
39	1	5.54034	-1.17087	1.18279
40	1	5.23155	0.5573	1.23709

Sum of electronic and zero-point Energies= -752.549680

Sum of electronic and thermal Energies= -752.529865

Sum of electronic and thermal Enthalpies= -752.528921

Sum of electronic and thermal Free Energies= -752.604257
